# The Replica Set Method is a Robust, Accurate, and High-Throughput Approach for Assessing and Comparing Lifespan in *C. elegans* Experiments

**DOI:** 10.3389/fragi.2022.861701

**Published:** 2022-04-28

**Authors:** Adam Cornwell, Jesse R. Llop, Peter Salzman, Niels Rasmussen, Juilee Thakar, Andrew V. Samuelson

**Affiliations:** ^1^ Department of Biomedical Genetics, University of Rochester Medical Center, Rochester, NY, United States; ^2^ Department of Biostatistics and Computational Biology, University of Rochester Medical Center, Rochester, NY, United States; ^3^ Department of Microbiology and Immunology, University of Rochester Medical Center, Rochester, NY, United States

**Keywords:** *Caenorhabditis elegans*, lifespan, survival modeling, biostatistics, high throughput

## Abstract

The advent of feeding based RNAi in *Caenorhabditis elegans* led to an era of gene discovery in aging research. Hundreds of gerogenes were discovered, and many are evolutionarily conserved, raising the exciting possibility that the underlying genetic basis for healthy aging in higher vertebrates could be quickly deciphered. Yet, the majority of putative gerogenes have still only been cursorily characterized, highlighting the need for high-throughput, quantitative assessments of changes in aging. A widely used surrogate measure of aging is lifespan. The traditional way to measure mortality in *C. elegans* tracks the deaths of individual animals over time within a relatively small population. This traditional method provides straightforward, direct measurements of median and maximum lifespan for the sampled population. However, this method is time consuming, often underpowered, and involves repeated handling of a set of animals over time, which in turn can introduce contamination or possibly damage increasingly fragile, aged animals. We have previously developed an alternative “Replica Set” methodology, which minimizes handling and increases throughput by at least an order of magnitude. The Replica Set method allows changes in lifespan to be measured for over one hundred feeding-based RNAi clones by one investigator in a single experiment- facilitating the generation of large quantitative phenotypic datasets, a prerequisite for development of biological models at a systems level. Here, we demonstrate through analysis of lifespan experiments simulated *in silico* that the Replica Set method is at least as precise and accurate as the traditional method in evaluating and estimating lifespan, and requires many fewer total animal observations across the course of an experiment. Furthermore, we show that the traditional approach to lifespan experiments is more vulnerable than the Replica Set method to experimental and measurement error. We find no compromise in statistical power for Replica Set experiments, even for moderate effect sizes, or when simulated experimental errors are introduced. We compare and contrast the statistical analysis of data generated by the two approaches, and highlight pitfalls common with the traditional methodology. Collectively, our analysis provides a standard of measure for each method across comparable parameters, which will be invaluable in both experimental design and evaluation of published data for lifespan studies.

## Introduction

**FIGURE 1:**
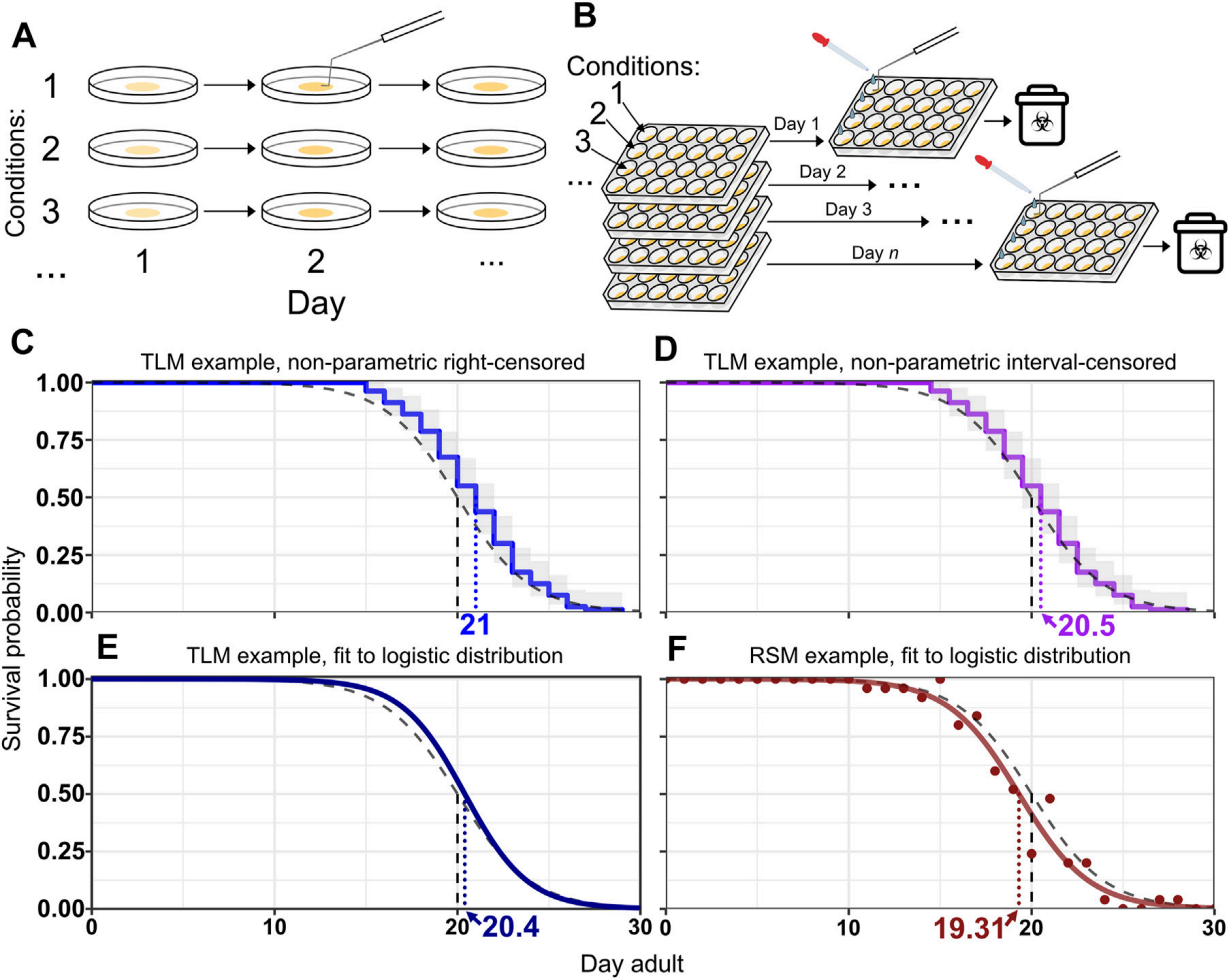
RSM and TLM assays employ different strategies for population sampling. **(A)** Handling in traditional lifespan assays (TLM). A dish of animals is maintained for the duration of the experiment. Vital status observation may be facilitated by prodding animals to stimulate movement. When more than one experimental condition is included, such as different strains or RNAi treatments, a separate plate is maintained throughout the experiment for each condition. **(B)** Handling in replica set lifespan assays (RSM). Each observation uses a different plate derived from a synchronized population, with all the plates for expected observations of the experiment having been set up at the beginning of the experiment. M9 solution is typically added to wells to separate animals from the bacterial lawn and to help assess vital status, in conjunction with prodding as necessary. While replica set assays can be done with single-well dishes as well, the approach is particularly well-suited to use of multi-well plates, in which wells of the plate can be different RNAi conditions. Every plate in the replicate group is set up with an identical layout. **(C–F)** Example survival curves from simulated lifespan experiments, assuming daily scoring. The black dashed curve represents the generating logistic distribution with mean/median of 20 days and shape parameter *s* = 2. The vertical dashed lines indicate the median for the respective curve. **(C–E)** A simulated TLM experiment was run with 80 animals and fit with Kaplan-Meier (KM) using right-censoring (blue curve) **(C)**, interval-censoring (purple line) **(D)**, or with parametric fitting to a logistic distribution (fit logistic curve is dark blue) **(E)**. The grey shaded region is the confidence interval for KM curves. **(F)** shows an example simulated replica set experiment using 25 animals per observation with the proportions of live animals for each observation shown as points, and the fit logistic curve, both in dark red. The median survival for the simulated TLM experiment is 21 days with right censoring **(C)**, 20.5 days with interval censoring **(D)**, and 20.4 days with parametric fitting **(E)**. The median survival for the simulated replica set experiment **(F)** is 19.3 days.

Aging is a gradual and progressive decline in physiological function, most prominently reflected in the rising probability of death over time for a given a population. Concordantly, lifespan is often used as a surrogate measure of aging as it is a straightforward and unambiguous measurement: an animal is either alive or dead. The traditional longitudinal method (TLM) of measuring lifespan in model organisms, including *C. elegans*, entails following a relatively small population of animals and tracking when death events occur. The *population* is observed at specific time points, and at each time point dead animals are counted and consequently removed (Figure 1A). Thus, the increasing mortality observed within a population is dependent on each previous measure of mortality. For example, if 5 animals within a population of 35 animals die on day 13, then there will be only 30 remaining animals to assess whether they are alive or dead at the next time point. Mortality within a chronologically age-matched population has been found to rise exponentially- with some natural variation between individuals- even within an isogenic population in a common environment, which cumulatively provides a distribution of how death events occurred within the population (Finch et al., 1990; Brooks et al., 1994).

### Traditional Assays to Follow *C. Elegans* Lifespan have a Number of Weaknesses

While using the TLM to measure lifespan in *C. elegans* is straightforward, it is relatively low-throughput, limiting the number of conditions that can simultaneously be measured. Comprehensive genome wide RNAi-based gene knockdown screens to date have identified in *C. elegans* 1147 putative gerogenes (broadly defined as genes whose function either extends or shortens longevity) (WormBase WS282). However, in many cases these gerogenes have been identified based on measuring viability at a single or a few time points (Lee et al., 2003; Hamilton et al., 2005; Hansen et al., 2005), which fails to provide a quantifiable measure of change in lifespan. Full longitudinal lifespan analysis is one prerequisite for identifying genetic interactions between gerogenes (*e.g.* epistatic, asynthetic interactions, etc.).

*C. elegans* viability is most often scored based on observable movement, which becomes less frequent and subtler as animals age. Young animals actively explore their environment and feed upon a lawn of *E. coli*, making scoring young animals straightforward. However, as an animal ages movement progressively declines, becoming increasingly uncoordinated and lethargic (Hosono et al., 1980; Bolanowski et al., 1981; Johnson, 1987; Herndon et al., 2002; Huang et al., 2004). Specifically, by day seven of adulthood, wild-type *C. elegans* may be observed that no longer display spontaneous active behavior in the absence of outside stimuli (Herndon et al., 2002; Huang et al., 2004). At more advanced age, increasing sarcopenia results in progressive degeneration of movement, ultimately resulting in paralyzed animals. Viability in an older animal is ascertained by observing subtle head movements at the very tip of the animal, increasing probability of scoring mistakes associated with progressing age (Duhon and Johnson, 1995; Herndon et al., 2002; Glenn et al., 2004; Gerstbrein et al., 2005). Additionally, scoring of the same population across multiple timepoints requires repeated handling, environmental exposure, and potential introduction of airborne contamination, which is not a trivial concern. For example, *C. elegans* lifespan can be altered by even subtle environmental changes: animals exposed to ambient light in a laboratory for as little as 20 min per day during scoring exhibit mean lifespan up to 12% shorter than animals scored in the dark (De Magalhaes Filho et al., 2018). Collectively, these drawbacks limit throughput, introduce variability, and increase the probability of experimental error with the TLM approach.

### The Replica Set Method

To overcome the limitations of the TLM, we previously developed an alternative experimental design to measure *C. elegans* viability that relies on the use of replica sets (Samuelson et al., 2007b, 2007a; Johnson et al., 2014; Cornwell et al., 2018; Cornwell and Samuelson, 2020). To this end, a large population of age-synchronized, isogenic animals are divided into a number of smaller samples (which we term as “replicas”) of approximately 15–20 animals each. Enough replicates are generated to cover each time point in the planned experiment. At each time point, one of the replicas is scored for the number of living, dead and censored animals, then discarded. Thus, over the period of time that covers the expected lifespan of the population as a whole, a series of independent subpopulations are sampled at each time point (Figure 1B). In using replica sets there is no repeated prodding of animals, and no repeated exposure to potential environmental contamination from opening and closing the same plates. The viability observed at one time-point is independent of every other observation, and hundreds of conditions can be tested in parallel– which increases throughput by at least an order of magnitude (for an example of increased throughput through application of the replica set method (hereafter RSM), see (Samuelson et al., 2007a)).

### Systematically Assessing the Performance of Replica Set and Traditional Methods

Understanding the nature of what each longevity assay measures and how each is analyzed is crucial; to that end we undertook a comparison of the accuracy, precision, and resilience between the methodologies using an *in silico* approach to contrast lifespan estimates obtained by both methods against a known standard. We show that the TLM is susceptible to an analysis-induced intrinsic bias in estimated median and mean lifespan, which is an artifact arising from the assumption that death occurred at the time of observation. Most of the widely used software to plot and analyze lifespan data-using the non-parametric Kaplan-Meier approach by default- assume death occurred at the time of observation, known as right-censoring (Figure 1C), rather than at an unknown time in the interval between observations, the latter of which is known as interval-censoring (Figure 1D) (Kaplan and Meier, 1958; Finkelstein, 1986). When observation intervals are consistent within an experiment, this bias does not affect the power to detect statistical differences, but is important when considering mean or median estimates of lifespan across experiments, or when the observation interval is varied between conditions to be compared within one experiment. We found that the most appropriate way to analyze data from a TLM experiment having discrete observation times with day-long (or longer) periods is to use interval censoring. We demonstrate that both TLM and RSM approaches have similar accuracy and precision in estimating lifespan using sample sizes and scoring frequency reflective of values from many published studies, when non-parametric analysis with interval censoring (Figure 1D) or parametric analysis (i.e. distribution-based curve fitting, in this case to the logistic distribution) is applied for TLM (see Figures 1E,F for examples of parametric analysis of TLM and RSM data, respectively). However, when several forms of scoring error are simulated, we discovered the estimated median generated through the TLM significantly deviates from the true median. In contrast, the RSM is robust to various experimental and measurement errors that are likely to be common in examination of *C. elegans* lifespan. Thus, the replica set method represents a resilient and high-throughput approach to quantitatively assess changes in *C. elegans* lifespan.

## Materials and Methods

### Lifespan Experiment Literature Survey and Assembly of a Gerogene Compendium

**FIGURE 2:**
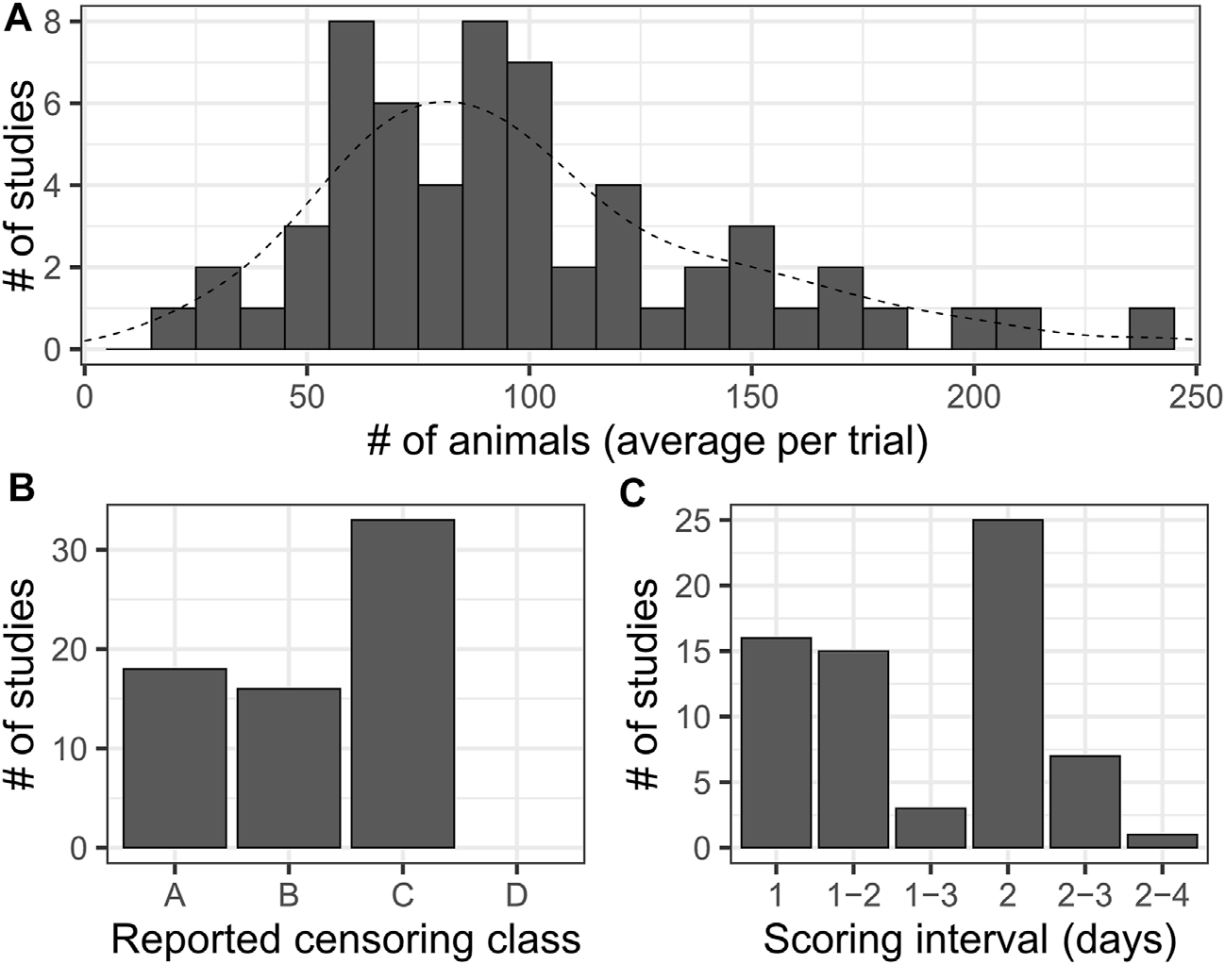
A Literature survey for lifespan studies using *C. elegans* highlights dramatic variation in sample sizes and levels of detail reported. **(A)** Histogram of average number of animals per trial for each study considered in a survey of literature reporting lifespan experiments for N2 (wild-type). Histogram bar width represents 10 animals. The dashed line is the smoothed density curve for the same data. Median: 90, mean: 99.46. This excludes two outlying data points, each reported once, that were unlikely representative of single-trial results (360 and 721 animals). **(B)** A summary of the level of detail reported on censored observations in the surveyed publications. We divided results into categories as follows: [A]- censoring was not mentioned in the manuscript and no censor information was reported in the data, [B]- Censoring was mentioned in the manuscript (*e.g.* “animals that crawled off plates were censored”), but no censor information was reported in the accompanying data, [C]- Censoring was mentioned in the manuscript and a summary of censored animal information (*e.g.* “12 animals were censored for trial 1”) was reported in the included data. [D]- Censoring was mentioned in the manuscript, and data for censored animals was provided for each time point of each trial. Note that we did not find an example of this last level among the literature surveyed. **(C)** Histogram of the reported scoring intervals from across the studies. Studies where a variable scoring interval was specified are indicated by a range of values. The most common scoring schedule was every-other-day scoring, indicated by an interval of two.

67 publications incorporating lifespan experiments with N2 animals were manually reviewed, spanning the years 2007–2020. For each paper, we noted the number of animals assayed per experimental trial (average across trials within a paper, when multiple unique values were provided), the interval between scoring occurrences, the lifespan and statistical analysis methods utilized, and the level of detail in reported censoring information. When scoring intervals were variable, the range was recorded (*e.g.* 2-3 for every 2 or 3 days). Reported censored observation information was classified into categorical bins A through D with increasing detail: A, no mention of censoring and no censored data reported; B, censoring mentioned but no censored data reported; C, censoring mentioned and censored observations were reported at a summary level (# of animals censored per condition or per trial); D, censoring mentioned and censored observations were reported per observation or timepoint including the reason for censoring (Figure 2C). In some cases, the complete details of the statistical analysis were not mentioned, but were able to be inferred, *e.g.* stating the log-rank test was utilized and showing stepped survival curves but not mentioning the Kaplan-Meier estimator. Papers for which it was not clear how the reported animal numbers were split into trials, which had a very high number of animals (1000+) were excluded from plots and summary calculations as such a large number of animals is unlikely to be derived from a single experimental trial of a single condition.

To establish the degree to which high-throughput lifespan studies have contributed to our knowledge of genetic interactions with lifespan in *C. elegans,* a collection of gerogenes and associated publications was assembled based on annotation curated in WormBase (Harris et al., 2009) version WS282 using the WormBase API through R version 4.0.2 (R Core Team, 2015). Genes, both coding and non-coding, were identified that were associated with one of the following phenotypes through RNAi or mutation experiments: “life span phenotype” (WBPhenotype:0000039), “shortened life span” (WBPhenotype:0001171), or “extended life span” (WBPhenotype:0000061). The citation information for the publications providing the evidence for the relationships were also retrieved.

### Parametric and Non-Parametric Fitting of Experimentally-Derived Lifespan Datasets

Four TLM experiments from two experimenters, six RSM experiments from two different experimenters, and five Lifespan Machine experiments from two different experimenters for N2 lifespan of animals kept at 20°C as adults were assembled from unpublished datasets. In order to obtain lifespan estimates, as well as determine the logistic curve shape parameter *s* from experimentally-derived datasets, TLM experiment data was fit with both parametric logistic and Kaplan-Meier approaches, RSM experiments were fit with the parametric logistic model, and non-parametric Lifespan Machine experiments were fit non-parametrically, all as described here under “Estimation of median, mean, and maximum lifespan”.

### Simulation of Lifespan Experiments

To simulate lifespan experiments, random virtual animals were generated from a probability distribution, either logistic or Gompertz, as indicated. Survival times were drawn from the distribution in batches, representing the population samples of cohabiting animals on plates (TLM) or wells (RSM) for lifespan experiments. For each batch of simulated animals, the corresponding number of survival times were independently drawn randomly. Each simulated trial was “scored” at time points corresponding to an interval of 1, 2, or 3 days which was fixed for a given trial. All simulation and evaluation work was performed in R version 4.0.2 (R Core Team, 2015).

For a TLM experiment, we simulated survival times on a single plate of N animals. This plate was then scored at each timepoint, with dead animals removed when they were “observed”, continuing for each increment in observation time until no animals were left “alive”.

For the RSM with N animals and T planned measurement timepoints, T plates of N animals each were drawn (N*T animals total). Analogous to real experiments, in which an estimate of expected maximum lifespan is obtained prior to setup of RSM trials, T was selected to include two observations past the expected maximum lifespan (99% survival of the generating distribution). For each time point, proportional survival was observed for a new “plate”, and each “plate” was scored once only. Scoring continued until reaching either two consecutive observations with no “live” animals, or until the prepared replicate plates (T) were exhausted. Across conditions of both methods, each experiment was repeated for 10,000 trials. Note that for two-sample testing and power analysis, 10,000 trials were generated but only 100 were considered due to constraints on computation time.

Reproducibility of these simulated experiments was facilitated by using a consistent pseudorandom number generator seeding strategy across all experiments. To generate simulated animals, a number of values from 0 to 1 was randomly drawn at uniform probability corresponding to the starting population sample size N (TLM) or the size of the population sample for each replicate N*T (RSM); these values were in turn used as quantiles to determine simulated animal death times based on the generating distribution. The quantile function for logistic is $T\left(p\right)=s*\ln\left(\frac{1}{p}-1\right)+\mu$ where T(p) is the time of death at quantile p , s is a parameter controlling curve shape, and μ is the mean/median. To represent survival of a wild-type population, we chose μ=20 and s=2 , with s derived from logistic curve fits to our own N2 lifespan data from both RSM and TLM assays, and mean/median of 20 days as a convenient approximation (Supplementary Figure S1).

To complete computation of the simulations in a reasonable time-frame, execution was parallelized to use multiple available cores on a desktop computer using the R package *future* or on a high-performance computing cluster (UR CIRC BlueHive) using the package *slurmR* (Yon and Marjoram, 2019; Bengtsson, 2021); animal populations were generated on the former, and permutation testing performed on the latter. To ensure reproducibility for generating animal population samples in parallel, the L'Ecuyer-CMRG random number generator was used for population generation, and the default R RNG was employed elsewhere for reduced computational overhead.

### Estimation of Median, Mean, and Maximum Lifespan

Each time a simulated plate was scored with either method, the time (day adult) and number of live and dead animals were recorded. For non-parametric analysis of TLM experiments, data was formatted with one record per individual with a death event. For right-censoring, which assumes the event occurred at the time of observation, only the observation time *t* and the death-indicating event code 1 were necessary. For interval-censoring, which assumes the event occurred between the observation at time *t* and the previous observation at *t-1*, the two times specifying the interval (*t*—1, *t*) were provided for each event. In both non-parametric analyses, the data was fit using the R package *survival* (Therneau, 2021) and the mean, median, and 95% quantile lifespan estimates were recorded for each simulated trial.

For analysis of RSM experiments, or parametric treatment of TLM data, the experiment result tables were converted to interval format, similar to TLM with interval censoring. For RSM, as each animal is only observed once and only the current status is known, an observed death at time *t* yields an interval of (0,t) , while animals observed alive have an interval of (t,∞) (*i.e.* we do not know when any given live animal eventually died). Parameters were estimated using non-linear minimization as implemented in the R function *nlm* (R Core Team, 2015). Specifically, $P\left(t\right)=\frac{1}{e^{u+1}}$ where $u=\frac{t-\mu }{s}$ with parameters µ (mean/median) and *s* (curve shape) selected to maximize likelihood of the observed survival intervals, calculated as P(right edge)−P(left edge) for each interval. The returned parameters were then used to find the 95% quantile lifespan estimate; mean and median lifespan are given by the parameter µ.

### Determining Accuracy and Precision Across Simulated Experiment Trials

10,000 simulated trials were performed for sample sizes of 5–50 for RSM and 5—150 for TLM, both in increments of 5 animals, for scoring intervals of 1, 2, and 3 days. For each combination of assay, scoring interval, population size, and analysis type, standard error (SE), and mean-squared error (MSE) were computed across the median lifespan estimates from the 10,000 trials as metrics of precision and accuracy, respectively. For daily scoring, sample sizes were identified that resulted in similar accuracy and precision across assay types, and these sample sizes were the focus of further analysis.

### Simulating Systemically Flawed Experiments and Fitting Data From a Different Generating Distribution

An experiment where two populations with differing lifespans are accidentally mixed was used as the motivating example for a case of a fundamentally flawed experiment which would not be able to produce the expected result with any lifespan assay methodology. To simulate this experiment, 67% of animals were generated with parameters *µ* = 20 and *s* = 2, and the remaining 33% of animals were generated with *µ* = 16 and *s* = 1.6, with the logistic distribution. Experiments for RSM and TLM were then run as described earlier.

To evaluate RSM performance when the generating distribution is different from the fitting distribution, simulations were performed where Gompertz was used as the generating distribution across the same set of assay types and analysis treatments as utilized elsewhere for single-sample experiments (*e.g.* experiment sample sizes, scoring interval). Experiment samples were generated as otherwise described for logistic, but with $T\left(p\right)=\frac{\ln(1-\frac{\beta }{\propto }\ln\left(1-p\right))}{\beta }$ for obtaining death times, corresponding to a two-parameter formulation of the Gompertz function (Benjamin, 1825; Pollard and Valkovics, 1992; Wilson, 1994). Selected parameter values were  ∝= 0.0003271342 and β= 0.3271342007 , such that median lifespan is 20 days and slope is comparable to logistic with μ = 20 and s = 2 (see curves in Figure 5A).

### Model for Experimenter Mis-Scoring and Scoring Hazard in Simulated Experiments

We simulated the effects of mis-scoring- either deeming a live animal as dead, or vice-versa- occurring at a probability, P(t) , which was either held constant, or modulated as a function of time. The probability of mis-scoring was modeled independently for each animal. In TLM experiments, mis-scoring affected which animals stayed on the plate or were removed: dead animals mis-scored as alive stayed on the plate to be scored again the following day, whereas live animals scored as dead were removed from the plate and never scored again. For the RSM, each animal was scored only once, and thus exposed to the possibility of being incorrectly scored only once.

We also simulated the possibility of formerly live animals being accidentally killed by the investigator and then recorded as dead through rough handling when making an observation- a scoring hazard. This type of error was simulated as a function of time only.

For constant-rate mis-scoring, a fixed error probability p was applied across the trial. When error rates were modeled as a function of time, the error rate started at zero, then rose to maxP according to the equation: $P\left(t\right)=\;maxP*\left(1-\frac{1}{e^{-\left(\frac{\mu -t}{s}\right)}+1}\right)$ where $u=\frac{\left(1.5t-\;\mu \right)}{s}$ , typically with μ = 20 and s = 2, or otherwise the same parameters as for the generating logistic distribution. Error probabilities p and maxP were set to 2, 4, or 10%. A set of results is provided for the case where the rise in error rate is inversely proportional to the decrease in survival at time t (“late-life onset” error), as well as for the biologically motivated scenario where error rate starts to increase in advance of mortality concomitant with the loss of mobility and onset of paralysis (“mid-life onset” error); the latter is obtained by using p(t∗1.5) to shift the error rate curve (Figure 6).

### Calculation of P-values and Power for Two-Sample Comparison Analyses

For analysis of statistical power, first 100 trials for RSM and TLM experiments were simulated across medians ranging from 16 to 24 days (+/- 20% from the reference of 20 days) in increments of 0.1 days all at population sample sizes for each trial from 5 to 50 animals for RSM and 5 to 150 animals for TLM. The generating distribution slope parameter was held at *s* = 2 for all cases. The result of a given trial was compared to the reference population (median of 20 days) with the same sample size and experiment type. *p*-values for comparison of parametric analysis for 100 generated experiment trials were computed using the R package *statmod* after 10,000 iterations of label permutation per trial as otherwise previously described (Phipson and Smyth, 2010; Cornwell et al., 2018). For non-parametric analysis, tests for differences in survival were performed with functions *survdiff* for right-censored data (Therneau, 2021), and *ictest* for interval-censored data (Fay and Shaw, 2010) for 100 generated trials for each relevant set of conditions. *p*-values were corrected for multiple testing across the *n* = 100 trial comparisons within a set of conditions using the Benjamini–Hochberg FDR adjustment (Benjamini and Hochberg, 1995). Power was then calculated as $power=\frac{\sum_{i=1}^{n}\begin{array}{cc} X_{i}<\propto & 1\\ X_{i}\geq \propto & 0 \end{array}}{n}$ for ∝=0.01 where i is the index in a vector of *p*-values X of length n .

## Results

### Previously Published Lifespan Studies Using Traditional Methods Vary Widely in Sample Size and Scoring Frequency

Among researchers measuring *C. elegans* lifespan using the traditional longitudinal method, a wide range of sample sizes and scoring periods have been reported. For example, one laboratory might survey a population of 50 animals for death events once per day. In contrast, another laboratory might measure survival within a population of 100 animals every third day. Thus, we compared and contrasted the accuracy and resiliency of the TLM and RSM across a wide range of scoring conditions. We posited that the sample size and scoring period have substantial effects on the accuracy and precision of lifespan estimates, thus we first surveyed the literature to identify the ranges actually used in published studies, and then modeled these *in silico* in order to identify how they altered the accuracy and precision of each method in estimating lifespan.

**FIGURE 3:**
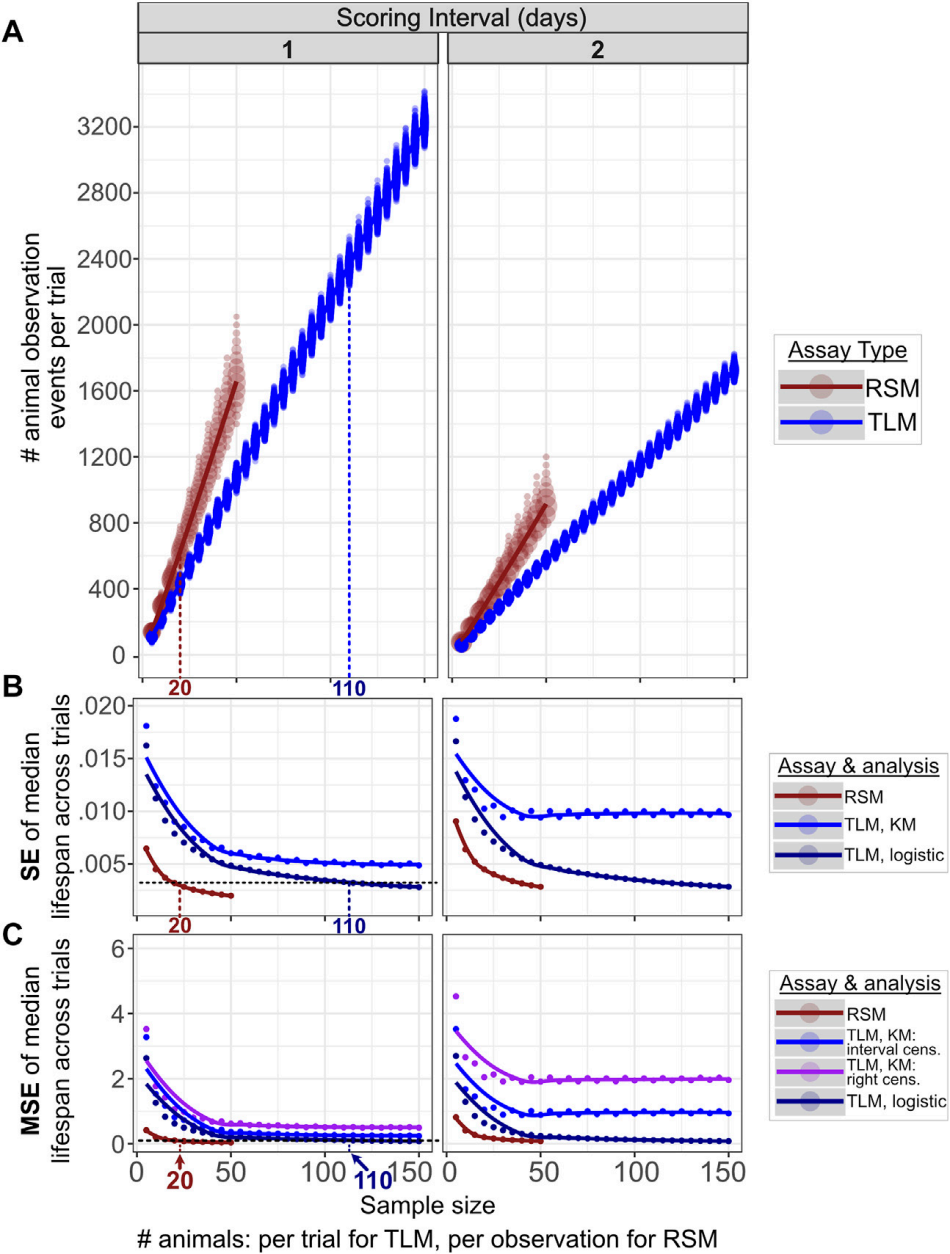
Comparable accuracy and precision can be obtained for RSM and TLM, but with many fewer total animal observations for RSM. 10,000 simulated RSM and TLM experiment trials were run for each increment in sample sizes across a range of 5–50 for RSM and 5—150 for TLM, both in increments of 5. For RSM, “sample size” refers to the number of animals per timepoint scored, whereas for TLM this is the number of animals at the start of the trial. The columns correspond to scoring intervals from 1 day (every day) or 2 days (every other day). We find fewer total animal observations are necessary to obtain a given level of precision (standard error of median lifespans and accuracy) **(B)**, (mean-squared error of median lifespans) **(C)** for RSM (median of 620 observations) compared to TLM (median of 2366 observations), with corresponding population sizes of 20 and 110 animals respectively. Increasing the interval between scoring timepoints negatively impacts both metrics for both assay types, particularly at small sample sizes. The same set of simulated TLM experiment data was used for all TLM analysis treatments. **(A)** The total number of animal observations (i.e. the number of times any animal is scored during the course of a trial, including live and dead animals, and those that have been scored on previous observations for TLM) scales differently with sample size between RSM (dark red) and TLM (blue) assays. “Sample size” is the number of animals per observation for RSM, and the number of animals per trial for TLM. The size of the circle at a given position indicates the number of trials which had that result. **(B)** SE (standard error) across the median lifespans for the 10,000 simulated lifespan trials for each sample size, for the given assay type and analysis method, indicating how precision improves with increasing sample size between the assay types. Right-censoring *vs*. interval-censoring shifts the medians but does not alter the dispersion, so only one set of results is shown for Kaplan-Meier analyses (denoted “KM”). For the left-most column (daily scoring) the horizontal black dashed line indicates a sample size for which SE is similarly low (0.003) across RSM and TLM (with parametric fitting to a logistic distribution, denoted “logistic”), 20 animals per observation for RSM and 110 animals per trial for TLM. **(C)** MSE (mean-squared error) across the median lifespans for the 10,000 simulated lifespan trials for each sample size, for the given assay type and analysis method, indicating how accuracy improves with increasing sample size between the assay types. For the left-most column (daily scoring), the horizontal black dashed line indicates a sample size for which MSE is similarly low (0.1) across RSM and TLM (with parametric fitting to a logistic distribution, denoted “logistic”), 20 animals per observation for RSM and 110 animals per trial for TLM.

To assess the sample sizes and scoring frequencies actually used in published research, we surveyed a representative sample of 67 manuscripts published between 2007 and 2020 incorporating *C. elegans* longevity estimates across a diverse array of high quality journals (Supplementary Table S1). We chose experiments using wild type animals, which have been reported to live between 16.6 and 20 days based on mean or median lifespan of N2 (wild-type strain) hermaphrodite animals maintained at 20°C (Kenyon et al., 1993; Gems and Riddle, 2000; Johnson et al., 2001, 2014). We assessed the number of animals included per experiment, the number of trials performed, the interval between scoring instances, and statistical aspects of the analysis (censoring, statistical software employed). Studies used a very broad range of values for the number of animals observed per experiment- from 10 to 1425 (mean = 115.2, median = 80) (Figure 2A). We also summarized the level of detail reported on censored observations- animals removed from an experiment due to alternative phenotypes or non-aging-associated death- on a scale from A (censoring not mentioned and no data reported) to D (censored animals reported for each time point) (Figure 3B) as high rates of censoring can impact the reported sample size of an experiment. Surprisingly, we did not find any examples of “class D” censoring among this set of publications, while nearly one quarter did not report any data on censored animals.

The interval between scoring was harder to assess, as some publications report variable scoring frequencies such as “every day to every other day”. Scoring frequency ranged from 1 to 4 days, with every other day being the most common observation interval (Figure 2C). From this we noted that the average interval between scoring instances is more than 10% of the median lifespan of wild-type animals, and sample size can vary by more than an order of magnitude. Thus, actual *C. elegans* lifespan studies using traditional methods vary widely in sample size and scoring frequency, which likely influences reproducibility and relative comparability between results.

### *In Silico* Modeling of Mortality and Accuracy of TLM and RSM Approaches

To evaluate the accuracy of the two methods in predicting median lifespan we first created a parametric model based on the logistic distribution from which to generate simulated populations of animals, with two parameters- one for mean/median and one for the shape/slope of the curve (denoted *s*). While the logistic function is symmetric, such that the mean and median are the same, for analysis in other contexts we focus on the median- the point at which 50% of the animals of a given population have died- as a summary value of lifespan in most cases. This “known standard” generating model represents an arbitrarily large theoretical population where the exact time of death for each animal is known. From this we then conducted an *in silico* analysis to determine how well each method captures the characteristics of our simulated standard. We set the slope parameter *s* = 2 for our generating model based on logistic curve fits to our own lifespan data across multiple experiments for wild-type (N2) *C. elegans* at 20°C with *E. coli* feeding on agar plates (Supplementary Figure S1), and assumed a mean/median of 20 days; this yields a generating distribution with mean, median, and 99% (maximum) survival of 20, 20 and 30 days, respectively.

To test how accurately and precisely the TLM and RSM approximate the median lifespan of the model population, we used a Monte-Carlo approach to generate 10,000 simulated experiment trials each for TLM and RSM (Dwass, 1957; Good, 2006), and calculated median as well as mean lifespan by both a parametric (i.e. fit to a distribution, in this case logistic) and non-parametric analysis (i.e. Kaplan-Meier) for TLM, and parametric analysis for RSM. Parametric analysis was performed for both assay types to ensure that the apparent benefits of RSM were not due to a difference in analysis methods; for both RSM and TLM this analysis was performed by finding parameters of a logistic curve which fit the data using non-linear minimization, as *C. elegans* lifespan data has been previously shown to fit a logistic distribution well (Vanfleteren et al., 1998). Concurrently, we assessed whether varying the sample size (5–50 animals per observation for RSM, 5—150 animals per trial for TLM) or scoring frequency (1–3 days) altered the accuracy or precision of either method.

### The TLM and RSM have Similar Precision and Accuracy in Predicting Median Lifespan, With RSM Requiring Fewer Total Animal Observations During the Course of an Experiment

To assess the precision of the experimental and analytical approaches, we investigated the variability resulting from the parametric and non-parametric analysis methods for TLM, and parametric analysis methods for RSM. The median lifespan estimates across experiment sizes for simulated daily scoring of TLM experiments had minimal differences in precision after both parametric and non-parametric analysis (as indicated by SEM) (Figure 3B). Increasing sample size (number of animals) substantially reduces variance in estimated average lifespan in both parametric treatments of the TLM, and, to a lesser extent, with RSM (Figure 3B). A similar trend is observed for the accuracy, as indicated by mean-squared error (MSE) of the median lifespan estimates (Figure 3C). It is worth noting that sample size is defined differently between the two methods- the TLM “sample size” reflects the total starting experiment sample size, whereas the RSM sample size represents the number of independent animals sampled at each time point- we sought to find experimental criteria for which the two methods were maximally comparable. We note that for daily scoring of TLM experiments starting with 110 animals per condition, the SEM and MSE of estimated lifespan is equivalent to an RSM experiment with 20 independent animals scored per condition (Figures 3B,C, respectively). Having demonstrated concordance between parametric and nonparametric models, we focus hereafter on simulation and comparison of TLM and RSM assuming 110 animals per condition for TLM, and 20 animals per observation for RSM, for daily scoring, with analyses based on the parametric approach. Finally, as these sample sizes are within the range of those commonly used in actual experiments, we conclude that the TLM and RSM methods have a similar level of precision and accuracy in estimating lifespan.

It must be noted that the number of observations necessary- *i.e.* the number of total animals observed during the course of an experiment- to obtain adequate power to detect a lifespan effect for each method is inherently different. As an example, in the TLM approach, for a given population, 110 individual animals may be observed in total, but in RSM 20 animals are measured per time point, with different individuals at each observation for the latter. The longitudinal nature of the TLM approach means that fewer measurements are made with progressing observations, until no animals are left alive. This is in contrast to an RSM experiment, where the number of measurements is the number of plates (*i.e.* time points) multiplied by number of animals per plate, with the number of animals remaining generally consistent. While the number of “unique animals” observed is much larger in the RSM experiment, the total number of animal observations- the number of times the researcher scores any animal per time point- is lower for RSM than TLM at a given level of precision and variance across simulated experiment trials (Figures 3A–C). As it takes time to determine if an animal is alive or dead, a reduction in the number of total animal observations indicates less time will be necessary to score the experiment. We find the nature of the assay for vital status in RSM, with the addition of liquid to a well to stimulate movement, further reduces the time needed to score an animal (Cornwell et al., 2018), and means that the net “hands-on” time is much shorter for a comparable RSM experiment.

### Analysis of TLM Data With a Right-Censoring Approach, But Not With Interval Censoring or Parametric Treatment, Leads to Biased Summary Survival Estimates

**FIGURE 4:**
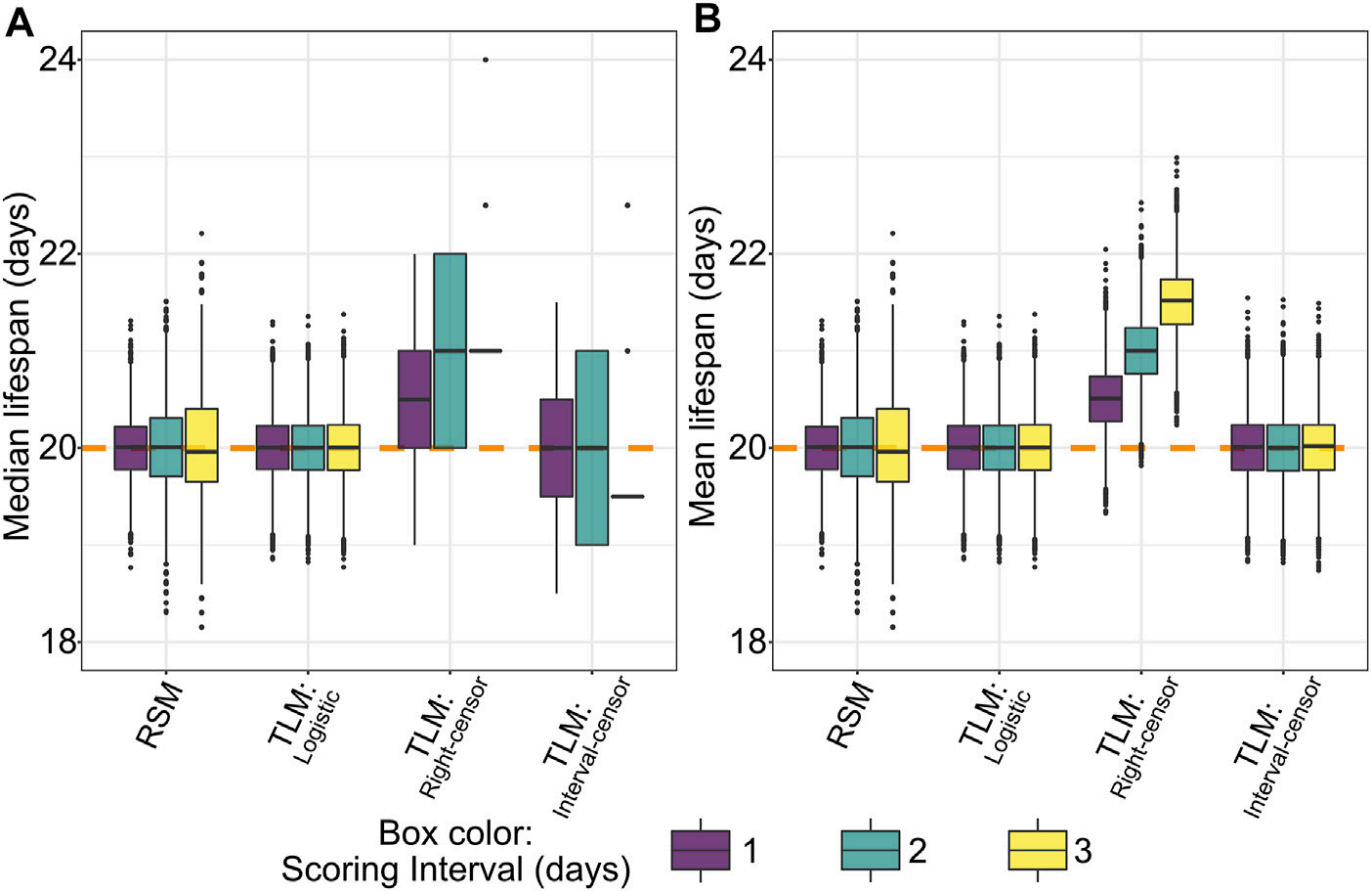
Non-parametric right-censored treatment of TLM data leads to right-edge bias, which is not observed with interval censoring, nor with parametric (logistic) fitting of TLM or RSM data. Across 10,000 simulated experiments for a sample size of 20 for RSM (# animals per observation) and 110 for TLM (# animals per trial) we find that non-parametric analysis of TLM data with right-censoring (denoted “Right-censor”) leads to a positive bias in estimated median **(A)** and mean **(B)** lifespan equivalent to approximately half the scoring interval. The dashed orange line indicates the mean/median of the generating logistic distribution (20 days). This bias is not observed in the TLM experiments analyzed with either a non-parametric treatment and interval censoring (denoted “Interval-censor”), or parametric treatment (logistic curve fit, denoted “Logistic”), nor with simulated RSM experiments and associated parametric analysis.

To our surprise, an initial comparison of parametric (logistic) treatment to a non-parametric (Kaplan-Meier) analysis of TLM-style data based on the simulated dataset revealed a bias in the non-parametric average lifespan estimates. This intrinsic bias increases the predicted median (Figure 4A) and mean (Figure 4B) lifespan by approximately one half of the scoring period (*e.g.* a bias of 0.5 days for daily scoring, 1.5 days for scoring every three days). The nature of this bias is a statistical artifact from assuming that death occurred at the time of observation, which is the case when right-censoring is employed- often the default for lifespan analysis software. To correct for this anomaly while maintaining a non-parametric analysis approach, one can use interval censoring, which places the time of death as occurring within an interval between the two observations. Correspondingly we found that the application of interval censoring eliminated this bias, as does parametric analysis of TLM data (Figures 4A,B).

Among the 67 papers that were part of our literature survey, we found that a Kaplan-Meier estimator was applied in 60 manuscripts (90%), but the use of interval censoring was not explicitly reported in any cases. In a brief investigation of recent versions of statistical software implementing survival analysis methods, we found some level of support (built-in or *via* community-derived scripts/packages) for interval censoring in nine out of 21 programs (Supplementary Table S2). Out of the representative literature we surveyed, only ∼25% utilized software that implemented interval censoring, regardless of whether it was utilized for the study (note that the older versions of the software used for the actual studies may not have supported interval censoring at the time); for about 23% of the studies considered we were not able to determine which statistical software, if any, was utilized. Thus, it is possible that right-edge bias, which is introduced by applying right-censoring instead of interval censoring, is likely to be prevalent in many reported *C. elegans* lifespan studies.

### Analysis of Replica Set Experiment Data With Logistic Curve Fitting Produces Reasonable Lifespan Estimates Even With Animals Drawn From a Different Distribution

**FIGURE 5:**
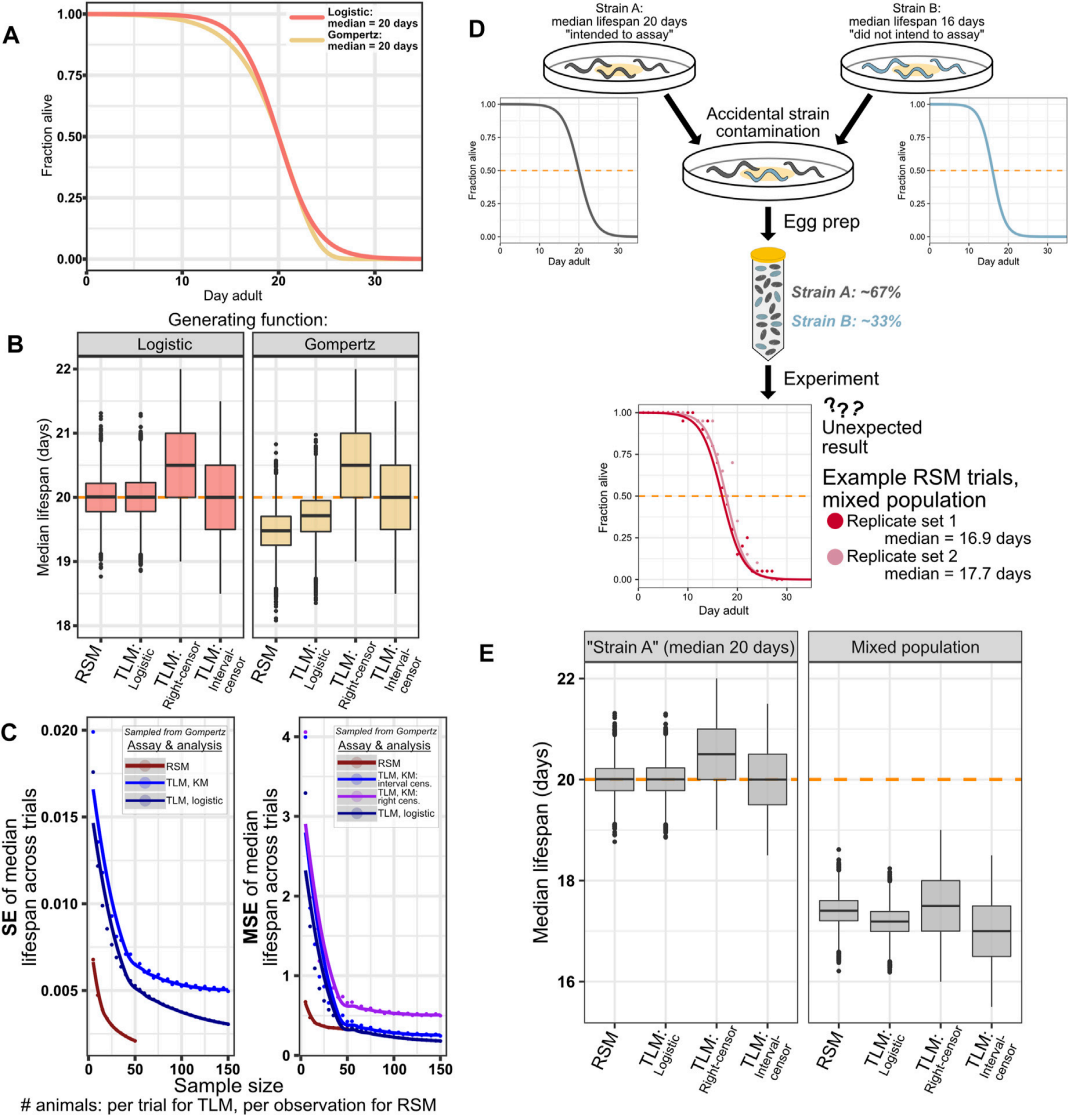
RSM shows reasonable performance even with populations generated from a distribution other than logistic. As expected, simulation of systemic errors in an experiment similarly impacts all assay and analysis types. **(A–C)** While *C. elegans* survival data has been shown to fit better to a logistic distribution than to Gompertz (Vanfleteren et al., 1998; Stroustrup et al., 2016), the possibility of treatments or genetic perturbations that can alter the shape of the curve cannot be dismissed. As such, we performed an experiment to ascertain how such a change in the generating distribution would affect the performance of the two assay types, while maintaining fitting to the logistic distribution for parametric treatments. **(A)** Animals were drawn from a logistic (orange) or Gompertz (yellow) distribution, both having median lifespan of 20 days. Parameters were chosen for the Gompertz curve such that the slopes were similar (see Materials and Methods). Note that unlike with logistic, the median and mean are not equal for Gompertz. **(B)** Median lifespan across 10,000 simulated lifespan trials for the indicated assay type and analysis treatment, for simulated animals with lifespan drawn from a logistic distribution (left) or Gompertz distribution (right). 20 animals per observation were used for simulated RSM experiments, and 110 animals per trial were used for the simulated TLM experiments. The input TLM experiment data was the same across the different analysis types. The “true median” of the generating distribution is indicated by the dashed orange line. As expected, non-parametric analysis of TLM experiments is not affected by the change in generating distribution, while the mismatch between the generating and fitting distributions results in a slight underestimate of lifespan for the RSM and parametric treatments of TLM- 19.48 and 19.71 days, respectively (median of the median lifespan across the 10,000 simulated trials, Supplementary Table S3). Precision is minimally affected by the change in distribution, as indicated by standard error (SE) of the median lifespans (**(C)**, left side), while mean-squared error (MSE) (**(C)**, right side) reflects the shift observed in **(B)** when compared to the similar plots of SE and MSE for the case of a non-mismatched distribution in Figure 3. **(D,E)** Systemic experimental errors, exemplified by a simulated case of mixing two *C. elegans* strains with different lifespans, affect all assay and analysis treatments similarly. **(D)** Schematic representation of a simulation of an experiment in which two strains with different lifespans are mixed. “Strain A” (dark gray) has median lifespan of 20 days, and represents the strain for which the assay might have been intended, but it becomes contaminated with “Strain B” (blue) which has substantially shorter lifespan of 16 days. The corresponding logistic curves show the generating distributions for these two populations. The result is a mixed population with 67% of animals having median lifespan of 20 days, and 33% of animals having median lifespan of 16 days, which yields a lifespan experiment result that is not the 20 days our experimenter expected (two example RSM experiment results from the mixed simulation are shown in red and light red). **(E)** Both assay types, and all TLM analysis approaches similarly reflect the result of the mixed experiment, yielding median lifespan estimates in between 16 and 20 days. 20 animals per observation were used for simulated RSM experiments, and 110 animals per trial were used for the simulated TLM experiments.

Although wild-type *C. elegans* lifespan has been previously shown to fit well to a logistic distribution (Vanfleteren et al., 1998), and a number of lifespan-modulating perturbations and treatments have been found to temporally scale lifespan in *C. elegans* rather than change the type of distribution (Stroustrup et al., 2016), we cannot exclude the possibility of encountering a condition that alters the shape of the survival curve. We posited that if we were to simulate survival experiments based on a different generating distribution, we would still obtain reasonable estimates of median survival from an RSM experiment with fitting the data to logisitic curves. To test this, we repeated our lifespan simulations, except with animal lifespan samples drawn from a Gompertz distribution, rather than a logistic distribution, while maintaining a median survival of 20 days (Figure 5A). We find that the change in generating distribution results in a small underestimate of lifespan for both the RSM and parametric treatment for TLM, a reduction in median lifespan of approximately 0.5 and 0.25 days, respectively (Figures 5B,C right side), while not changing the dispersion of the estimates across the simulated trials (Figure 5C left side). As expected, altering the shape of the generating distribution while maintaining the same median did not affect the estimates for non-parametric treatment of TLM experiments (Figure 5B). Thus, even when the experiment data does not match the assumptions of our logistic-based parametric analysis, we find that RSM still provides reasonable estimates of lifespan-with greater accuracy than the non-parametric right-censored analysis of TLM- without loss of precision.

### Assessing Effects of Scoring and Experimental Error

In addition to biological variation due to intrinsic stochastic factors within a single population of animals (Herndon et al., 2002), a lifespan experiment may suffer from extrinsic error, such as: incorrect scoring of individual viability, strain contamination, incubator temperature fluctuation, mating, insufficient food availability, or extended exposure to blue spectrum light (Gems and Riddle, 1996; De Magalhaes Filho et al., 2018; Baugh and Hu, 2020). We systematically investigated some of these extrinsic sources of variability by adding error terms to our simulations of both RSM and TLM experiments.

#### Systemic Experimental Error Simulation

For an error that shifts the expected survival of every animal by an equal amount, independent of measurement parameters (*e.g.* observation frequency, sample size) and methods (TLM or RSM), we would expect equal effects on the outputs from both methods. This would occur in a real experiment if, for example, there was a contaminating strain leading to a mixed population, the wrong strain was used in one comparative trial, or RNAi was inefficient. To this end, we simulated a mixed population case such that a third of the animals were short-lived by 20% (median lifespan of 16 days) compared to our standard population at 20 days (Figure 5D). We posited that this would shift the median lifespan estimates similarly across assay and analysis types. Indeed, we found that for either TLM or RSM there was a congruent decrease in estimated lifespan (Figure 5E). Thus, we can conclude that systemic error has a consistent effect across experimental methodologies, and regardless of the data analysis strategies considered for TLM.

#### Mis-Scoring Error Simulation

We next modeled how scoring error- inaccurately determining whether an animal is alive or dead- affected the accuracy and precision of the output of each method. We posit that as animals age and lose the ability to respond to stimuli through body-wall muscle movement, there is an increased probability that an experimenter will make a mistake in scoring viability. As errors in scoring can propagate through the remaining time-course for TLM but not RSM, we hypothesized that such scoring errors would introduce both more variability and bias in TLM compared to RSM experiments.

**FIGURE 6:**
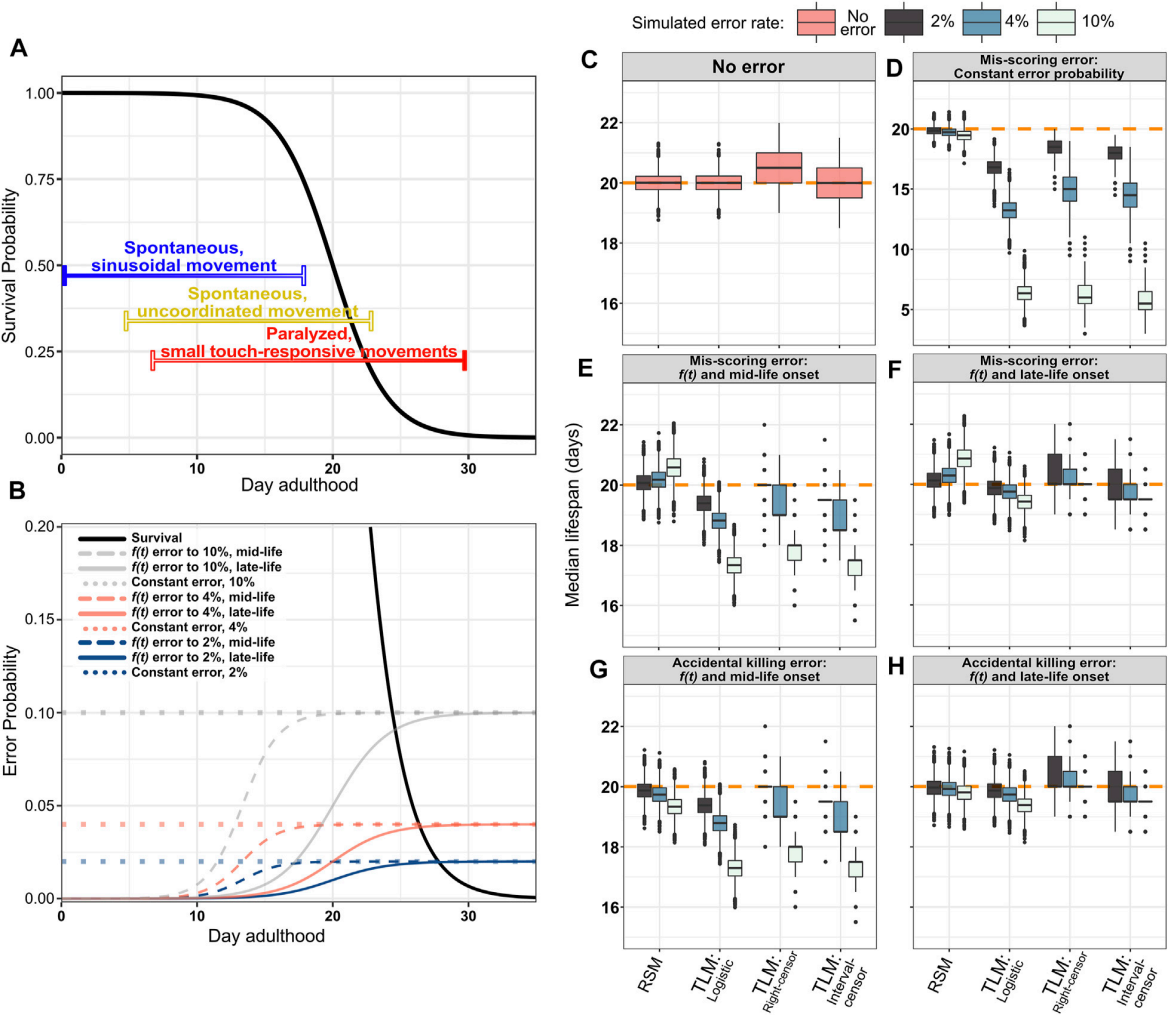
The RSM is more resilient to incorrect scoring of survival, as well as premature animal death caused by accidentally rough handling. As animals age, they demonstrate locomotory decline and increased fragility, making them more difficult to score for viability, and increasing the possibility of scoring errors to which RSM is more robust. **(A)** An illustration of the phases of movement and the decline thereof during *C. elegans* lifespan (Huang, C., Xiong, C., and Kornfeld, K. (2004), Newell Stamper, Breanne L., et al. (2018)). Young animals move spontaneously and frequently, with a consistent sinusoidal motion (indicated by blue bar). For scoring lifespan assays, it is usually quickly determined if animals in this stage are alive, as any that have temporarily stopped moving can be stimulated by gently tapping the plate. In “middle aged” animals, movement becomes less coordinated and frequent, but they still may be moving without any external stimulus (indicated by yellow bar). A stronger plate tap, or occasional prod with a pick or fiber may occasionally be necessary to stimulate movement. In aged animals, spontaneous translational body movement has largely ceased, with only occasional posture shifts of the head or tail (indicated by red bar). In this stage, it is usually not immediately evident if an animal is alive or dead, and touch stimulation is usually necessary to determine their vital state. As this can be laborious, the possibility of making an error in determining the state of an animal may increase, as does the chance of accidentally killing animals that were actually alive in the process of poking them to stimulate movement. Even in isogenic *C. elegans* populations in identical conditions housed on a single plate, there may be substantial variability between the movement states of different animals, represented as the color gradients of the bars. **(B)** Simulated error probability curves shown in relation to the generating logistic distribution (black solid curve) for single-sample experiments (mean/median = 20 and *s* = 2). Error rates were simulated at a fixed probability or as a function of time. Constant error rates were set at either 2, 4, or 10% (dotted lines). Modeling error as a function of time more closely mimics the increased likelihood that an experimenter will make a mistake as animals become paralyzed or fragile with age. The hazard function from the logistic distribution is used to increase the error probability as animals age, starting from either mid-life (dashed lines) or late-life (solid colored lines) reaching the maximum (2, 4, or 10%) near the end of life. The constant error and 10% error scenarios are not intended as a simulation of likely error rates during actual experiments, but serve to demonstrate the degree to which RSM improves robustness to accidental scoring and handling errors under extreme conditions. **(C–H)** Median lifespan across 10,000 simulated RSM and TLM trials for the indicated error models and analysis treatments, with a sample size of 20 animals for RSM (per observation) and 110 for TLM (per trial), and assuming daily scoring. The orange dashed line indicates the mean/median of the generating logistic distribution (20 days). **(C)** No simulated error. **(D)** Constant probability of mis-scoring. **(E,F)** Mis-scoring with probability modeled as function of time, rising from 0% to the indicated maximum rate starting in mid-life **(E)** or late-life **(F)**. **(G,H)** Simulated accidental death, in which an animal which was actually alive is accidentally killed by rough handling and subsequently called as dead, with probability modeled as function of time, rising from 0% to the indicated maximum rate starting in mid-life **(G)** or late-life **(H)**.

We sought to test this hypothesis through three simulation conditions: a constant mis-scoring error rate throughout lifespan, mis-scoring that begins at mid-life, and mis-scoring that begins late in life. For each condition we simulated error-rates of 2, 4, and 10%. The first condition serves as a proof of principle positive control to demonstrate whether incorrect assessment of viability affects the perceived lifespan by either RSM or TLM (Figure 6B, dotted lines). As expected, a constant probability of incorrect scoring resulted in both a loss of precision (i.e. increased variability) and accuracy in estimating lifespan with the TLM. Specifically, introduction of a constant 2% mis-scoring error yielded a median lifespan estimate of 16.8 days; shorter than the actual median lifespan of 20 days by 15.9% (with parametric analysis) (Figure 6D, Supplementary Table S3). In contrast, the same error applied to the RSM resulted in no appreciable change in accuracy (19.85 days, a decrease of 0.75%) (Figure 6D, Supplementary Table S3). If we increase the mis-scoring rate to 10%, the accuracy of both assays suffers, but the difference in bias is stark: the TLM median lifespan is 6.36 days (with parametric analysis), while RSM is 19.46 days (Figure 6D). It may be observed that the TLM estimates from non-parametric analysis with right-censoring appear to be less compromised with respect to accuracy than the other TLM treatments, but this is incidental due the aforementioned right-hand bias which yields an overestimate of lifespan under non-error conditions (Figures 6C,D). Thus, we conclude that the TLM is more sensitive to scoring error than the RSM, and shows a downward-shifted bias when even a small scoring error is introduced.

We next sought to determine how incorrect scoring would affect accuracy in a more realistic context, taking into account the behavioral changes that occur during aging in *C. elegans.* Assessing whether an individual is alive or dead typically relies on observing animal movement for both experimental approaches (TLM and RSM). As *C. elegans* animals age, they undergo a progressive decline in movement, which ultimately results in paralysis of most of the body in the last 3rd of an animal’s lifespan for WT (Hosono et al., 1980; Bolanowski et al., 1981; Johnson, 1987; Herndon et al., 2002; Huang et al., 2004) (Figure 6A). From the onset of paralysis, it becomes necessary to determine the status of animals from subtle head movement, often only in response to touch- making scoring much more difficult and time-consuming. Conversely, it is easy to determine whether an animal is alive or dead early in life, as movement is frequent and spontaneous (Figure 6A). To simulate the increase in scoring difficulty that follows this progressive decline in movement with age, we simulated scoring error- either false positive, or false negative- with probability rising from 0% for young animals up to 2, 4, or 10% later in life. The timing of the rise in the error rate was also modeled in two ways. The first simulates a “mid-life onset”, which represents incorrect scoring of animals starting from the decline in movement that occurs in advance of the highest rate of mortality, but within the period of time when a sub-set of individual wild-type animals have been empirically observed to enter a decrepit state (Herndon et al., 2002) (Figure 6B, dashed lines). This condition simulates a population where the vast majority of animals will still be responsive to touch, and therefore scored correctly, but a small subset of paralyzed animals within the larger population will be incorrectly scored at a higher rate. Even if the maximal probability of making such a mistake is just one in 50 animals, the shift in accuracy for TLM is nearly ten times that of RSM, at 19.4 days compared to 20.07 days, respectively (Figure 6E, Supplementary Table S3). This difference is exacerbated further when assaying long-lived animals, as just a 2% mis-scoring rate increasing from mid-life reduced median survival results from TLM by approximately two days when populations were generated from a distribution based on *daf-2(e1370)* lifespan characteristics (median 42 days, maximum 60 days (Kenyon et al., 1993; Bansal et al., 2015; Hahm et al., 2015; Podshivalova et al., 2017; Zhao et al., 2021)) (Supplementary Figure S3). When the possibility of mis-scoring increases to a maximum of 10%, the TLM estimate dips to 17.34 days (Figure 6E). Interestingly, we find that the RSM lifespan estimate actually increases- to 20.58 days- this is attributable to a difference in when RSM experiments are ended. Whereas TLM experiments end when there are no animals left alive from the starting population, typically an RSM experiment is terminated when no live animals are observed on two consecutive observations; consequently, mis-scoring a dead animal as alive near the end of the experiment could “reset the clock” and extend the experiment if the termination condition is strictly enforced, as it was for these simulations.

We also simulated a “late-life onset” of mis-scoring which represents incorrect scoring of animals that are entirely paralyzed or dead (Figure 6B, solid colored lines). In this scenario, the difference in accuracy between the assays appears to be less stark, which may be due to the smaller population remaining in the TLM experiments by the time the rate of mis-scoring peaks; when the possibility of mis-scoring rises to 10% starting from mid-life, the median TLM estimate is 19.43 days (Figure 6F). Here again, we observe an apparent over-estimate in lifespan for the RSM, attributable to the termination condition in the RSM simulations being reset when “dead” animals are scored as “live” near the end of the experiment. Thus, RSM provides a more accurate estimate of lifespan when the possibility of mistaking if an animal is alive or dead starts to increase near the time when movement begins to decline, even when the rate of such errors is low.

#### Age-Associated Fragility Hazard Simulation

During aging, *C. elegans* become increasingly fragile, making them more susceptible to injury by physical handling. As such, an intrusive method of scoring vital status could damage or possibly kill older animals, affecting their survival. Typically, older animals are gently prodded with the end of a platinum wire to induce movement, which could damage or kill those animals if too much force is accidentally applied. Therefore, we introduced an increasing probability of “killing” the animal in the process of scoring, from 0% and increasing to a 2, 4, or 10% starting from either mid-life (Figure 6B, dashed lines) or late-life (Figure 6B, solid colored lines). We found that introducing even a small probability of investigator-induced accidental death of an animal late in life, such that only one in 25 animals is affected (4%), resulted in a decrease in estimated median lifespan by 1.3% when followed through the TLM (parametric analysis); in contrast, the RSM estimated lifespan decreased by only 0.4 and 0.97% for a late life scoring hazard of 4 and 10%, respectively (Figure 6H, Supplementary Table S3). As expected, if the onset of frailty- and the associated increase in hazard-is shifted to mid-life, the TLM again yields dramatically shorter lifespan estimates, from 19.38 days with 2% hazard rate to 17.29 days with 10% hazard rate, compared to 19.33 days for RSM even at the 10% rate (Figure 6G, Supplementary Table S3). Thus, the lifespan estimates of a population of *C. elegans* determined through the TLM is much more prone to error than the RSM if the process of scoring vital status of an animal impairs survival.

### RSM Requires Fewer Total Animal Observations to Obtain Adequate Statistical Power in Comparing Lifespan Across Conditions

**FIGURE 7:**
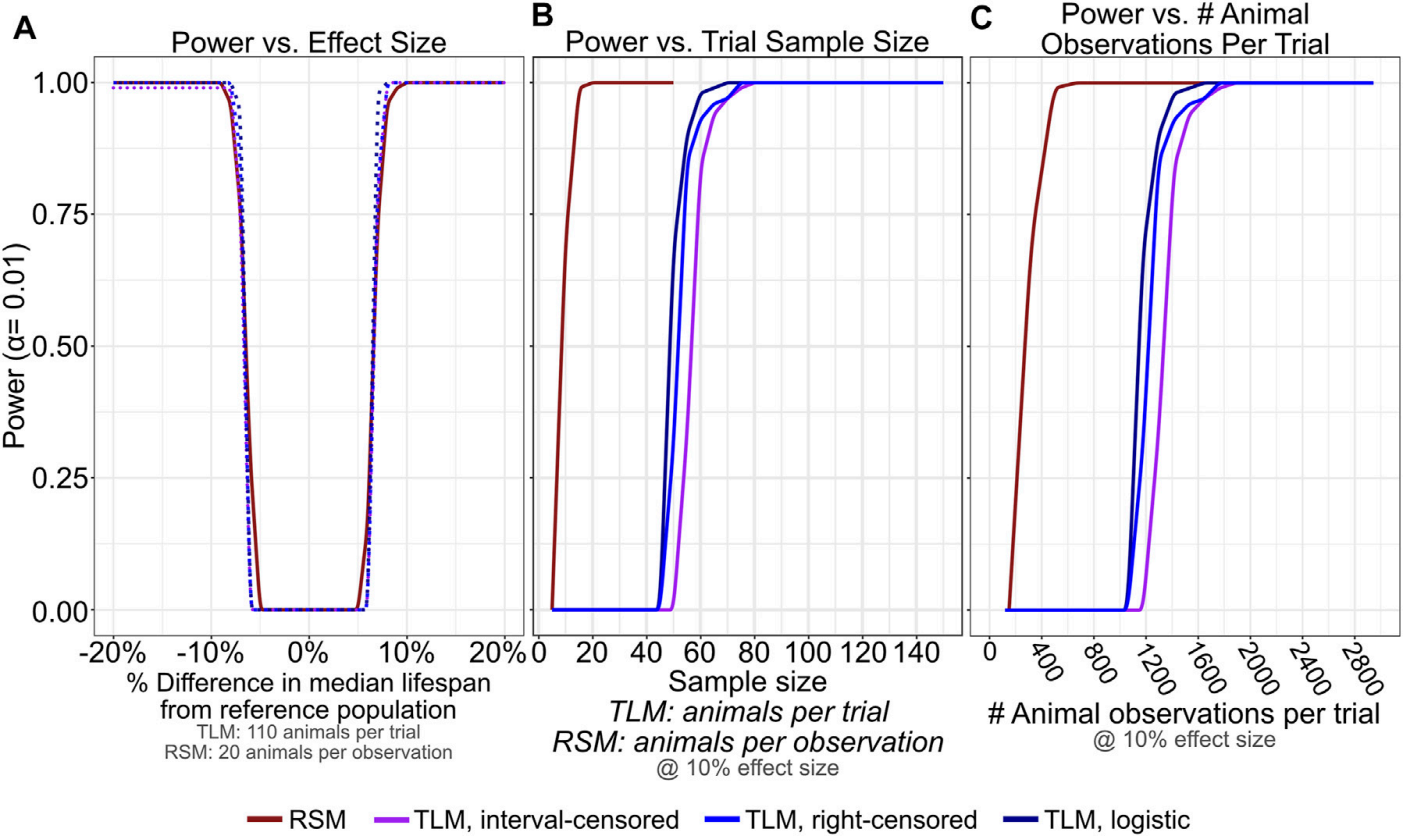
RSM provides similar statistical detection power to TLM, with many fewer necessary animal observations. 100 lifespan experiment trials were simulated for RSM and TLM each for every combination of sample size (5–50 per observation for RSM, and 2–150 per trial for TLM, both in increments of 5) and generating distribution mean/medians from 16—24 days (in increments of 0.1 days), all assuming daily scoring. For a given sample size and assay/analysis type, each trial was compared using an appropriate testing strategy against a reference population generated from a distribution with mean/median of 20 days. *p*-values determined from these tests were corrected for multiple testing (FDR) within the set of likewise trials and used to calculate power at alpha level of 0.01. **(A)** Statistical detection power is comparable across RSM and all analysis treatments of TLM for a broad range of effect sizes when considering 20 animals per observation for RSM and 110 animals per trial for TLM. **(B,C)** For an effect size of 10% (+/- 2 days compared to the reference population) RSM saturates power at a number of animals per observation **(B)** that corresponds to a far smaller number of total animal observations per trial compared to TLM **(C)**. The analysis treatments of TLM experiments all perform similarly when the scoring interval is held constant.

In aging research, comparative lifespan has been widely used between many types of conditions to ascertain if a statistically significant change has occurred between conditions (e.g. mutant versus wild-type animals, et. cetera). In order to determine how the TLM and RSM approaches influence the power of detection between two samples, we performed additional simulations, varying the median of the generating distributions (−20% to +20% in 0.1 day increments with respect to a reference population with median survival of 20 days) across a range of sample sizes (5–50 animals for RSM, and 5 to 150 animals for TLM) with daily scoring. We calculated power assuming an alpha of 0.01 after computing *p*-values for the comparison of 100 simulated trials against the reference population with all other conditions held constant, using the log-rank test and label permutation for non-parametric and parametric analyses, respectively. When we focus on the sample sizes previously determined to yield similar accuracy and precision between the assays for single-sample estimates of lifespan (Figure 3)- 110 animals per trial for TLM, and 20 animals per observation for RSM- we find that statistical power is also broadly comparable for the assays and analysis approaches across the range of effect sizes tested (Figure 7A). To see how the TLM and RSM differ in the amount of investigator effort necessary to achieve sufficient statistical power for a moderate difference in lifespan, we looked at the number of total animal observations per trial when the effect size was held at+/-10% (Figure 7C). The RSM experiments actually require fewer than half the number of total observations as the TLM to reach a power of 1, indicating that a corrected *p*-value < 0.01 was obtained for all 100 trial comparisons. We next repeated these comparisons, but with experiments for which mis-scoring error had been simulated, to determine how mistakes in scoring could end up influencing statistical power differently depending on the chosen type of lifespan assay. We see that even with a moderate rate of mis-scoring- up to a rate of 4% rising from late-life (Figure 6B, solid orange curve)- RSM power of detection remains robust, again with many fewer total observations necessary than TLM (Supplementary Figure S2). Overall, this corroborates our empirical experience with RSM enabling more conditions to be scored in the same amount of time as a given TLM experiment, without compromising the interpretability or utility of the results.

## Discussion

### The Replica Set Method is Distinct From the Traditional Longitudinal Method

We demonstrate that the TLM approach for assessing lifespan is less accurate and precise, as well as less resilient to several forms of error compared to the RSM. Furthermore, the RSM yields comparable statistical power with many fewer necessary total observations. This arises from the fundamental difference in their physical setup, and the statistical implications of the structure of each method. The survival observed at any time point using the TLM is dependent on the survival observed at each previous point. In contrast, observations of animal survival in the RSM are completely independent. It is this independence that helps reduce both intrinsic and extrinsic variability in the RSM.

We find that the intrinsic variability, accuracy, and statistical power of an TLM experiment with a sample size of 110 is comparable that of the RSM with a sample size of 20. Taking these values as an example, if one were to sum the total number of times the vital status of any animal is determined over the course of an experiment by both methods, with daily scoring, the RSM would generate about 600 observations and the TLM would produce about 2300 observations (Figure 3). However, in the RSM viability of 600 distinct animals is measured, while the TLM would only have measured mortality of 110 unique animals. Consequently, the RSM measures more individual animals, while the TLM makes more total observations. These two parameters have different weights between the two methods: a single additional animal at the start of a TLM experiment adds more observations than a single animal added to an observation of an RSM experiment. In contrast, increasing *sample size* by one animal produces not only more observations in the RSM, but each of those added measures is more informative as each observation is independent. This asymmetry helps explain why increasing sample size reduces RSM variability- and increases statistical power- more quickly than it would with TLM. Thus, real TLM and RSM data with a sample size of 110 and 20 animals, respectively, have similar precision in estimating lifespan.

### The Traditional Method Suffers Bias due to Right-Censoring

An artificial increase in estimated lifespan as a function of the scoring interval has been previously observed for *C. elegans* TLM experiments using simulation (Petrascheck and Miller, 2017). In concordance with their finding, we showed that the TLM has an intrinsic bias that increases estimated mean and median lifespan by approximately one half of the scoring period (e.g. bias of 0.5 days for once-daily scoring) (Figures 4A,B). This is due to scoring frequency: daily or alternate day scoring makes a discrete, periodic measurement of survival. However, survival is a continuous phenotype, thus the time between actual and observed death creates a bias, with magnitude directly proportional to the time between survival measurements. This is not evident when observation intervals are dramatically shortened- as in the case of automated experiment platforms such as the Lifespan Machine (Stroustrup et al., 2013)- or in studies involving longer-lived organisms where the scoring precision is very fine relative to the total lifespan.

Importantly, we find that analysis of the same experiment data non-parametrically but with interval censoring, or parametrically by logistic curve fitting, abrogates the right-censoring-induced bias. Rather than assuming an event occurred at the time of observation as with right-censoring, interval-censoring places the time of death in the middle of the interval between observations (Gomez et al., 2009; Zhang and Sun, 2010). Our simulations show very little mean or median bias when interval censoring is applied, up to a scoring interval of 3 days, which we find is on the upper end of the interval actually used across a range of *C. elegans* survival studies (Figure 2B). Fitting the same simulated TLM experiments to logistic curves- similar to the analysis approach taken with RSM data- also avoids inducing the bias we observe in the right-censored non-parametric analysis (Figures 4A,B). As the bias represents a shift in the mean and median survival estimates, the dispersion of the data is not affected, and there is minimal effect on statistical power between right-censoring and interval-censoring when comparing groups where observations were made with the same observation schedule (Figures 7A,B). In contrast, if the groups compared were observed at variable intervals (“every 2–3 days”), or if scoring was performed at different intervals between the groups (*e.g.* every day for group 1, alternate days for group 2), this right-censoring bias would influence the results, possibly producing false positive or negative conclusions. Thus, it is critical to consider interval censoring or parametric analysis when designing experiments or reviewing disparate experimental approaches. In our literature survey of 67 papers including N2 lifespan experiments, we found no cases where interval censoring was utilized (Supplementary Table S1), despite support for such analysis being available in some common statistical software packages such as SPSS, STATA, JMP, and R (Supplementary Table S2). Similarly, in considering the information on analysis software collected from 824 publications including N2 lifespan experiments in a recent meta-analysis (Urban et al., 2021), more than 40% of publications used software that does not presently support interval censoring- much of which is accounted for by Graphpad Prism alone- an indication that there is not yet awareness of the benefits of interval censoring for *C. elegans* survival studies.

In contrast to the TLM, the RSM makes a discrete measure of a discrete phenotype: each day an independent sample of individuals is counted, and each animal is either alive or dead at this time. With RSM we are indifferent to the timing of death outside of this single time point; instead we measure proportional survival, a quantity unbiased by scoring time.

### The Traditional Method is Less Resilient to Error

The TLM is less resilient then the RSM in accurately estimating mean and median lifespan of a population when even a small probability of incorrectly scoring viability later in life is introduced. The difference in response to scoring error stems entirely from the fact that dead animals are removed from the plate once observed in TLM. If a live animal is incorrectly scored as dead, its lifespan is artificially shortened at least one day, but potentially more as individual decrepit and frail animals can remain alive for an extended period of time (Johnson, 1987; Herndon et al., 2002). Conversely, if a dead animal is accidentally scored as live, it is likely to be properly scored dead at the next time point. Thus, TLM scoring error is heavily biased towards death. In contrast, the RSM measures an individual animal once, thus incorrectly scoring viability affects only a single observation, and is consequently more robust to such bias.

In addition to the increasing difficulty with age in determining whether an animal is alive or dead, aging *C. elegans* become increasingly fragile. Touching animals with a platinum wire and looking for movement is a common technique for applying a stimulus intended to cause a locomotory response in animals that are alive but not otherwise moving. Loss of touch responsiveness to light touch in aged animals makes scoring viability more difficult, and may promote the use of a more forceful touch stimuli that kills a decrepit animal. While it is difficult to know the actual hazard rate that occurs from poking animals without a non-damaging approach to ascertain true live/dead status, for simulation we chose maximal hazard rates of 1 in 50 (2%), 1 in 25 (4%), and 1 in 10 (10%) intuitively (Figures 6G,H). The actual rate of animal death from damage due to touch will be highly dependent on the individual conducting the experiment. Surprisingly, when we apply a fragility hazard rising with time from around mid-life, the median lifespan from the TLM is decreased by 3.1% compared to 0.7% for RSM at a maximum of 2% error, and up to 15% for TLM compared to just 3.3% for RSM at a maximum of 10% error, when both assays were treated parametrically (Figure 6G). An alternative touch response approach to using a typical platinum wire would be to use an eyelash or similar fiber, which is less damaging to aged animals (Sulston and Horvitz, 1977). If a live animal is killed by touching it in the process of stimulating movement, in the TLM the error propagates to affect multiple measurements. Importantly, like mis-scoring, the RSM is also resilient towards this sort of error. This makes sense intuitively, as the scoring hazard does not accumulate. Collectively, the independence of individual measurements in the RSM insulates it from errors introduced by incorrect scoring or repeated handling of increasingly fragile animals.

### Limitations of Both Methods for Assessing Lifespan

The TLM and RSM for scoring lifespan have distinct limitations. For instance, throughput is a major limitation to the TLM; scoring a sample by the RSM can be done much faster, for several major reasons. First, addition of liquid to the wells in the RSM acts as a mild stimulus that causes animals to swim, which aids in discriminating live animals from dead ones. Furthermore, sarcopenia in older animals limits movement particularly in the viscous bacterial lawn, and the addition of liquid in the RSM scoring approach helps to liberate the animals. Second, as the RSM insulates the experimenter from propagating scoring errors, it allows one to more quickly make decisions on whether an individual animal is alive or dead without concern of how a mistake would influence the rest of the experiment. Third, each well of the multi-well plates used for RSM are small, such that the entire well can fit in a microscope field of view, which makes for faster tracking of the whole population. In contrast, the Petri-style plates commonly used for TLM are too large to view the entire population at one time, which requires scanning the whole plate. While RSM experiments can involve a large number of plates, the required initial setup time may be reduced by employing common laboratory aids such as multi-well electronic pipettes. If incubator space is found to be limiting, particularly relevant for experiments in long-lived mutant backgrounds or where an intervention is expected to extend lifespan, the number of plates may be reduced by planning to score less frequently until signs of aging- such as movement decline- are observed. It is worth noting that specific equipment and software to facilitate the use of the RSM can be found described in: (Cornwell et al., 2018; Cornwell and Samuelson, 2020). A limitation inherent to the RSM is that progeny production must be prevented: either through the use of fluorodeoxyuridine (FUdR), which in some cases produces lifespan phenotypes that can confound analysis (Aitlhadj and Stürzenbaum, 2010; Van Raamsdonk and Hekimi, 2011), or sterile genetic mutants. However, some genetic interactions that influence lifespan are only revealed when progeny production is actively inhibited. For example, it was long thought that the TGFβ pathway did not regulate *C. elegans* aging (Kenyon et al., 1993; Larsen et al., 1995), however, the use of FUdR revealed that TGFβ signaling regulates longevity through insulin signaling (Shaw et al., 2007). The link between TGFβ signaling and longevity was missed without the use of FUdR, as TGFβ pathway mutations produce a slight egg laying defect (*egl*) and extend reproductive longevity, which causes internal hatching of progeny later in life that kills the parent prematurely. Of note, starved wild-type animals also manifest an *egl* phenotype, perhaps as an adaptive survival advantage to progeny under conditions of low food (Baugh and Hu, 2020); this has implications for studies on the genetics of dietary restriction (DR), which may be complicated by overlap between models of DR and starvation responses (Mair and Dillin, 2008). Thus, using either a chemical inhibitor or genetic mutant to limit progeny production versus manually transferring animals are both valid; each has advantages and disadvantages, and is one aspect to consider when choosing a methodology.

The type and amount of the bacterial food source is another important consideration in planning either type of lifespan experiment. Starvation must be avoided for accurate assessment of lifespan (Johnson et al., 1984), which is typically managed by adjusting the bacterial volume added to a well or plate, the number of animals added, and by concentrating bacterial cultures if necessary. While using FUdR to inhibit reproduction obviates the need to transfer parents away from progeny that will quickly deplete the available food, it is still possible to accidentally distribute too little bacterial culture or too many animals to a given plate or well, making for a higher probability of a single well “starving out”. In TLM, this is avoided by keeping spare plates prepared at the same time as the others, but without animals added, onto which the animals are manually transferred from the at-risk plate- a process which is prone to error, particularly when animals are aged and fragile, and can introduce contamination. For RSM, this is avoided by preparing extra replicate plates at the outset of the experiment- which also have animals added like all other plates in the set- which can be scored as necessary to replace a plate with starved wells (Cornwell et al., 2018; Cornwell and Samuelson, 2020). Starved wells are easy to identify and are censored. Additionally, the common bacterial food sources used in *C. elegans* culture have been found to differ in nutritional composition and influence developmental rate and lifespan of *C. elegans* (Stuhr and Curran, 2020). Thus, with either lifespan method care must be taken to ensure consistency in food source (*e.g.* strain and concentration), and proactive preparation of spare plates or replicates will reduce the possibility of having to discard or restart a trial due to starvation.

### TLM, RSM, and Lifespan Assay Automation

Improvements in affordable imaging hardware as well as machine learning have led to an increasing number of options for automating *C. elegans* longitudinal assays such as lifespan. Most such systems utilize cameras and record images at intervals or video, and use software to identify animals and determine the time at which movement ceases. Present approaches represent different positions across a balance between throughput and detail on the activity of individual animals: full-motion video is necessary to reconstruct movement tracks or record activity levels across the entire adult life of animals, but these systems are difficult to scale to thousands of animals or conditions (*e.g.* the Nemalife Infinity system, SiViS), while periodic imaging is suitable for constructing a time-lapse across life of many more animals or conditions, but is not able to track the movement path of individual animals early in life when used with multiple plates (*e.g.* the Lifespan Machine, WormBot) (Stroustrup et al., 2013; Pitt et al., 2019; Rahman et al., 2020; Puchalt et al., 2021). Others utilize alternative experiment or measurement paradigms in order to approach the middle of the balance, such as the Phylum Arena system (PhylumTech, Santa Fé, Argentina), which uses an array of infrared microbeams to detect movement of animals rather than traditional imaging, and WorMotel (Jushaj et al., 2020) which is based on singling animals in microplate-sized wells, thus removing the need for continuous video to build a life history of activity for individual animals. While all of these systems track the same population of animals across time, the scoring approaches are passive, nondestructive, and generally obviate the need to re-expose animals to the environment after the start of the experiment, thereby reducing exposure to airborne contaminants similar to RSM experiments. However, mechanically stimulating movement- as with adding liquid to a well in RSM or poking with a wire- may be advantageous when working with animals harboring mutations that increase the time spent in a behaviorally quiescent sleep-like state, such as *daf-2* mutants, which, if unstimulated, can cease movement for periods long enough to be called as dead by the default movement analysis models that are bundled with the Lifespan Machine software, in our experience (Gaglia and Kenyon, 2009; Stroustrup et al., 2013). These hardware platforms may also be unsuitable for chronic heat tolerance assays, as this could require elevating the temperature of the surrounding environment or enclosure and thus also the instruments themselves. The “set it and forget it” nature may also lead to mistakes that are not caught until the planned end of the experiment- starvation, progeny, contamination, *etc*- unless the researcher checks for anomalies periodically. Large-scale automation of lifespan experiments still involves substantial upfront costs, such that RSM remains an attractive option for applications such as RNAi library screening across large gene families or across the genome. Even when automated approaches are available, investigators might consider running a manually-scored TLM or RSM trial alongside the automated experiment, thereby acquiring two trials worth of data, with the hands-on time of just one.

### Statistical Analysis of the Traditional Longitudinal and Replica Set Methods

At the completion of a lifespan experiment, it is important to determine the mean, median, and maximum lifespan within a sample, as well as to identify significant differences in lifespan between conditions of interest. For TLM experiments, summary statistics of lifespan can be estimated using the Kaplan-Meier estimator. The Kaplan-Meier estimator is a non-parametric statistical estimator frequently applied to lifespan data because it provides a number of benefits for survival analysis applicable to many studies in research and clinical settings (Kaplan and Meier, 1958). First, the Kaplan-Meier estimator accounts for one-sided censoring of animals; censoring is important in situations where an animal must be removed from study (*i.e.* died for reasons other than aging). For instance, in the case of *C. elegans,* animals that crawl up the side of the plastic dish will desiccate and die. In these cases, it is known approximately how long the animal lived before the event that warranted censoring, but when the animal would eventually have died due to aging is unknown. The non-parametric nature of Kaplan-Meier removes the necessity of verifying that the data is appropriate for a given distribution- there are very few assumptions to satisfy. However, parametric survival functions- which require ensuring that the data is a good fit to the chosen distribution (often Gompertz, logistic, or Weibull)- are also available for longitudinal lifespan data, and may be more accurate for cases when the surviving fraction approaches zero at the termination of the study (Miller, 1983; Wilson, 1994; Mudholkar et al., 1996).

To determine whether there are significant differences in lifespan between two populations when using Kaplan-Meier analysis, the Mantel-Cox log-rank test is applied (Mantel, 1966). This statistical test has been a standard within the field for many years and allows one to identify significant differences in mean/maximum lifespan. The log-rank test is non-parametric, and takes censoring into account (Bland and Altman, 2004). A popular alternative to the log-rank test is the Wilcoxon test, which is also non-parametric, but whereas log-rank has been found to be biased toward differences near the end of the experiment timeline (*i.e.* termination of the study), Wilcoxon can be biased toward differences at the beginning (Tarone, 1981).

The RSM yields current status data, representing a form of interval censoring in which every data point is either left-censored (for animals that are dead at time of observation) or right-censored (for animals that are alive at time of observation). As the basic Kaplan-Meier estimator does not handle current status data, calculation of mean, median, and maximal lifespan of data generated through the replica set method must be determined by other methods. While non-parametric methods for handling current status data have been described and implemented, this remains an active area of development. In particular, the nonparametric maximum likelihood estimate (NPMLE) (Gomez et al., 2009) as implemented in the R packages “Interval” (Fay and Shaw, 2010) or “icenReg” (Anderson-Bergman, 2017) may provide a basis for nonparametric analysis of RSM data. For the present work, we chose to model this data parametrically using the logistic distribution, as *C. elegans* lifespan has previously been shown to fit this distribution well (Vanfleteren et al., 1998). Consistently, we have also previously found that large lifespan datasets derived from the RSM fit best to a logistic model, but can also fit a Gompertz distribution (Samuelson et al., 2007b; 2007a), the latter of which is frequently used for demographic analysis of mortality in mammals (Finch and Pike, 1996). Parametric and nonparametric methods of survival analysis have been shown to perform similarly when the data is a good fit for the chosen distribution (Efron, 1988).

Identifying significant differences in lifespan between two populations through the RSM is determined by first fitting the data from a given condition to a logistic model. The median lifespan values are calculated from these model fits; logistic curves are symmetrical about their inflection point, such that the median is equal to the mean (Winsor, 1932). The *p*-values for the desired comparison are then computed using a Monte Carlo resampling approach using 10,000 iterations to obtain precise *p*-values (Robinson, 2007; Phipson and Smyth, 2010). Confidence intervals for the median lifespan values can also be derived from the Monte Carlo simulation. Although these results can take some time to compute for a large number of comparisons, it is trivial to parallelize for significant speedup on most modern computers.

Our analysis of power to detect differences for traditional lifespan is concordant with previous findings using simulations and non-parametric analysis (Petrascheck and Miller, 2017). Additionally, both studies highlight the importance of adequate sample size for detection of a given magnitude difference in lifespan for traditional longitudinal experiments, and identify that accuracy is compromised with longer scoring intervals in traditional experiments. We further highlight that scoring frequency can influence accuracy and precision in determining mean and median lifespan, and that analysis of TLM experiments with interval censoring may produce estimates of longevity that are more comparable between studies. Importantly, we show that Replica Set experiments require relatively few animals per observation to obtain similar or better detection power compared to TLM experiments (Figure 7). Furthermore, our results demonstrate that scoring errors- simulating an incorrect assessment of animal vital status or accidentally damaging fragile aged animals during scoring- even at low probability and introduced late within an experiment, can drastically influence lifespan estimates for TLM assays.

We demonstrate that the RSM is more accurate, precise, and robust to error then the TLM for estimating lifespan in *C. elegans.* A lack of repeated handling reduces exposure to airborne contaminants, and removes possible error from prodding of increasingly fragile aging animals. Furthermore, the RSM is amenable to measuring lifespan at high throughput and at low cost. Lifespan of several hundred test conditions (e.g. feeding based RNAi) can be simultaneously followed, allowing one to hasten the discovery of genetic interactions and pathways (Samuelson et al., 2007a). Furthermore, when applying the RSM to *C. elegans*, liquid is added to the well at the time of scoring, which not only increases scoring accuracy, but also provides a stimulus for spontaneous movement, reducing the need for time-consuming manual touch stimulation (Cornwell et al., 2018; Cornwell and Samuelson, 2020). Through video capture of thrashing/swimming *C. elegans* it is possible to quantify activity, using pixel displacement (A.V.S. unpublished observations), to obtain an estimate of healthspan (i.e. the period of life that animals actively respond to stimuli, see (Samuelson et al., 2007a)). Thus the RSM can easily and simultaneously track both survival and health during aging. Collectively this study highlights the limitations and pitfalls of the TLM for measuring lifespan of *C. elegans* and provides, in the RSM, an accurate, precise, high-throughput alternative that is less susceptible to common types of experimental errors.

## Data Availability

The R source code and simulated datasets used for this study will be made available at https://github.com/samuelsonlab-urmc or from the authors by reasonable request.

## Supplementary Material

Supplementary Figure S1 N2 (WT) lifespan experiment data examples for RSM, TLM, and an automated lifespan platform (the “Lifespan Machine”).

Supplementary Figure S2 Even with moderate rates of mis-scoring, RSM provides statistical power of detection similar to TLM, with many fewer total animal observations necessary.

Supplementary Figure S3 RSM is more robust than TLM to errors from incorrect scoring of survival in in simulated experiments based on long-lived animals.

Supplementary Table S1
*C. elegans* lifespan assay literature survey.

Supplementary Table S2 A survey of statistical software supporting lifespan analysis.

Supplementary Table S3 Summary values (median and mean) across single-sample simulated lifespan experiments.

## References

[B1] Aitlhadj L. Stürzenbaum S. R. (2010). The Use of FUdR Can Cause Prolonged Longevity in Mutant Nematodes. Mech. Ageing Development 131, 364–365. 10.1016/j.mad.2010.03.002 20236608

[B2] Anderson-Bergman C. (2017). icenReg: Regression Models for Interval Censored Data in R. J. Stat. Soft. 81. 10.18637/jss.v081.i12

[B3] Bansal A. Zhu L. J. Yen K. Tissenbaum H. A. (2015). Uncoupling Lifespan and Healthspan in *Caenorhabditis elegans* Longevity Mutants. Proc. Natl. Acad. Sci. U.S.A. 112, E277–E286. 10.1073/pnas.1412192112 25561524 PMC4311797

[B4] Baugh L. R. Hu P. J. (2020). Starvation Responses throughout the caenorhabditis Elegans Life Cycle. Genetics 216, 837–878. 10.1534/genetics.120.303565 33268389 PMC7768255

[B5] Bengtsson H. (2021). A Unifying Framework for Parallel and Distributed Processing in R Using Futures. R. J. 13, 208. 10.32614/rj-2021-048

[B6] Benjamin G. (1825). On the Nature of the Function Expressive of the Law of Human Mortality, and on a New Mode of Determining the Value of Life Contingencies. Philos. Trans. R. Soc. Lond. 115. 10.1098/rstb.2014.0379 PMC4360127 25750242

[B7] Benjamini Y. Hochberg Y. (1995). Controlling the False Discovery Rate: A Practical and Powerful Approach to Multiple Testing. J. R. Stat. Soc. Ser. B (Methodological) 57, 289–300. 10.1111/j.2517-6161.1995.tb02031.x

[B8] Bland J. M. Altman D. G. (2004). The Logrank Test. BMJ 328, 1073. 10.1136/bmj.328.7447.1073 15117797 PMC403858

[B9] Bolanowski M. A. Russell R. L. Jacobson L. A. (1981). Quantitative Measures of Aging in the Nematode *Caenorhabditis elegans*. I. Population and Longitudinal Studies of Two Behavioral Parameters. Mech. Ageing Dev. 15, 279–295. 10.1016/0047-6374(81)90136-6 7253717

[B10] Brooks A. Lithgow G. J. Johnson T. E. Lithgow G. J. (1994). Mortality Rates in a Genetically Heterogeneous Population of *Caenorhabditis elegans* . Science 263, 668–671. 10.1126/science.8303273 8303273

[B11] Cornwell A. B. Llop J. R. Salzman P. Thakar J. Samuelson A. V (2018). The Replica Set Method: A High-Throughput Approach to Quantitatively Measure *Caenorhabditis elegans* Lifespan. Jove J. Vis. Exp. 29, e57819. 10.3791/57819 PMC6102014 30010651

[B12] Cornwell A. B. Samuelson A. V. (2020). “Analysis of Lifespan in *C. elegans*: Low- and High-Throughput Approaches. Methods Mol. Biol. 2144, 7–27. 10.1007/978-1-0716-0592-9_2 32410021 PMC9113156

[B13] De Magalhaes Filho C. D. Henriquez B. Seah N. E. Evans R. M. Lapierre L. R. Dillin A. (2018). Visible Light Reduces *C. elegans* Longevity. Nat. Commun. 9. 10.1038/s41467-018-02934-5 PMC5834526 29500338

[B14] Duhon S. A. Johnson T. E. (1995). Movement as an index of vitality:Comparing Wild Type and the Age-1 Mutant of *Caenorhabditis elegans* . Journals Gerontol. Ser. A: Biol. Sci. Med. Sci. 50A, B254–B261. 10.1093/gerona/50A.5.B254 7671016

[B15] Dwass M. (1957). Modified Randomization Tests for Nonparametric Hypotheses. Ann. Math. Statist. 28, 181–187. 10.1214/aoms/1177707045

[B16] Efron B. (1988). Logistic Regression, Survival Analysis, and the Kaplan-Meier Curve. J. Am. Stat. Assoc. 83, 414–425. 10.1080/01621459.1988.10478612

[B17] Fay M. P. Shaw P. A. (2010). Exact and Asymptotic Weighted Logrank Tests for Interval Censored Data: TheintervalRPackage. J. Stat. Soft. 36, 1–34. 10.18637/jss.v036.i02 PMC4184046 25285054

[B18] Finch C. E. Pike M. C. (1996). Maximum Life Span Predictions from the Gompertz Mortality Model. Journals Gerontol. Ser. A: Biol. Sci. Med. Sci. 51A, B183–B194. 10.1093/gerona/51A.3.B183 8630694

[B19] Finch C. E. Pike M. C. Witten M. (1990). Slow Mortality Rate Accelerations during Aging in Some Animals Approximate that of Humans. Science 249, 902–905. 10.1126/science.2392680 2392680

[B20] Finkelstein D. M. (1986). A Proportional Hazards Model for Interval-Censored Failure Time Data. Biometrics 42, 845–854. 10.2307/2530698 3814726

[B21] Gaglia M. M. Kenyon C. (2009). Stimulation of Movement in a Quiescent, Hibernation-like Form of *Caenorhabditis elegans* by Dopamine Signaling. J. Neurosci. 29, 7302–7314. 10.1523/JNEUROSCI.3429-08.2009 19494152 PMC2919683

[B22] Gems D. Riddle D. L. (2000). Defining Wild-type Life Span in *Caenorhabditis elegans* . J. Gerontol. A. Biol. Sci. Med. Sci. 55, B215–B219. 10.1093/gerona/55.5.b215 10819307

[B23] Gems D. Riddle D. L. (1996). Longevity in *Caenorhabditis elegans* Reduced by Mating but Not Gamete Production. Nature 379, 723–725. 10.1038/379723a0 8602217

[B24] Gems D. Sutton A. J. Sundermeyer M. L. Albert P. S. King K. V. Edgley M. L. (1998). Two Pleiotropic Classes of Daf-2 Mutation Affect Larval Arrest, Adult Behavior, Reproduction and Longevity in *Caenorhabditis elegans* . Genetics 150, 129–155. 10.1093/genetics/150.1.129 9725835 PMC1460297

[B25] Gerstbrein B. Stamatas G. Kollias N. Driscoll M. (2005). *In Vivo* spectrofluorimetry Reveals Endogenous Biomarkers that Report Healthspan and Dietary Restriction in *Caenorhabditis elegans* . Aging Cell 4, 127–137. 10.1111/j.1474-9726.2005.00153.x 15924569

[B26] Glenn C. F. Chow D. K. David L. Cooke C. A. Gami M. S. Iser W. B. (2004). Behavioral Deficits during Early Stages of Aging in *Caenorhabditis elegans* Result from Locomotory Deficits Possibly Linked to Muscle Frailty. Journals Gerontol. Ser. A: Biol. Sci. Med. Sci. 59, 1251–1260. 10.1093/gerona/59.12.1251 PMC1458366 15699524

[B27] Gómez G. Calle M. L. Oller R. Langohr K. (2009). Tutorial on Methods for Interval-Censored Data and Their Implementation in R. Stat. Model. 9, 259–297. 10.1177/1471082X0900900402

[B28] Good P. I. (2006). Resampling Methods: A Practical Guide to Data Analysis. 10.1007/0-8176-4444-X

[B29] Hahm J.-H. Kim S. DiLoreto R. Shi C. Lee S.-J. V. Murphy C. T. (2015). *C. elegans* Maximum Velocity Correlates with Healthspan and Is Maintained in Worms with an Insulin Receptor Mutation. Nat. Commun. 6, 8919. 10.1038/ncomms9919 26586186 PMC4656132

[B30] Hamilton B. Dong Y. Shindo M. Liu W. Odell I. Ruvkun G. (2005). A Systematic RNAi Screen for Longevity Genes in *C. elegans* . Genes Dev. 19, 1544–1555. 10.1101/gad.1308205 15998808 PMC1172061

[B31] Hansen M. Hsu A.-L. Dillin A. Kenyon C. (2005). New Genes Tied to Endocrine, Metabolic, and Dietary Regulation of Lifespan from a *Caenorhabditis elegans* Genomic RNAi Screen. Plos Genet. 1, e17–0128. 10.1371/journal.pgen.0010017 PMC1183531 16103914

[B32] Harris T. W. Antoshechkin I. Bieri T. Blasiar D. Chan J. Chen W. J. (2009). Wormbase: A Comprehensive Resource for Nematode Research. Nucleic Acids Res. 38, D463–D467. 10.1093/nar/gkp952 19910365 PMC2808986

[B33] Herndon L. A. Schmeissner P. J. Dudaronek J. M. Brown P. A. Listner K. M. Sakano Y. (2002). Stochastic and Genetic Factors Influence Tissue-specific Decline in Ageing *C. elegans* . Nature 419, 808–814. 10.1038/nature01135 12397350

[B34] Hosono R. Sato Y. Aizawa S. I. Mitsui Y. (1980). Age-dependent Changes in Mobility and Separation of the Nematode *Caenorhabditis elegans* . Exp. Gerontol. 15, 285–289. 10.1016/0531-5565(80)90032-7 7409025

[B35] Huang C. Xiong C. Kornfeld K. (2004). Measurements of Age-Related Changes of Physiological Processes that Predict Lifespan of *Caenorhabditis elegans* . Proc. Natl. Acad. Sci. U.S.A. 101, 8084–8089. 10.1073/pnas.0400848101 15141086 PMC419561

[B36] Johnson D. W. Llop J. R. Farrell S. F. Yuan J. Stolzenburg L. R. Samuelson A. V. (2014). The *Caenorhabditis elegans* Myc-Mondo/Mad Complexes Integrate Diverse Longevity Signals. Plos Genet. 10, e1004278. 10.1371/journal.pgen.1004278 24699255 PMC3974684

[B37] Johnson T. E. De Castro E. Hegi de Castro S. Cypser J. Henderson S. Tedesco P. (2001). Relationship between Increased Longevity and Stress Resistance as Assessed through Gerontogene Mutations in *Caenorhabditis elegans* . Exp. Gerontol. 36, 1609–1617. 10.1016/S0531-5565(01)00144-9 11672983

[B38] Johnson T. E. (1987). Aging Can Be Genetically Dissected into Component Processes Using Long-Lived Lines of *Caenorhabditis elegans* . Proc. Natl. Acad. Sci. U.S.A. 84, 3777–3781. 10.1073/pnas.84.11.3777 3473482 PMC304959

[B39] Johnson T. E. Mitchell D. H. Kline S. Kemal R. Foy J. (1984). Arresting Development Arrests Aging in the Nematode *Caenorhabditis elegans* . Mech. Ageing Development 28, 23–40. 10.1016/0047-6374(84)90150-7 6542614

[B40] Jushaj A. Churgin M. Yao B. De La Torre M. Fang-Yen C. Temmerman L. (2020). Optimized Criteria for Locomotion-Based Healthspan Evaluation in *C. elegans* Using the WorMotel System. PLoS One 15, e0229583. 10.1371/journal.pone.0229583 32126105 PMC7053758

[B41] Kaplan E. L. Meier P. (1958). Nonparametric Estimation from Incomplete Observations. J. Am. Stat. Assoc. 53, 457–481. 10.2307/228186810.1080/01621459.1958.10501452

[B42] Kenyon C. Chang J. Gensch E. Rudner A. Tabtiang R. (1993). A *C. elegans* Mutant that Lives Twice as Long as Wild Type. Nature 366, 461–464. 10.1038/366461a0 8247153

[B43] Larsen P. L. Albert P. S. Riddle D. L. (1995). Genes that Regulate Both Development and Longevity in *Caenorhabditis elegans* . Genetics 139, 1567–1583. 10.1093/genetics/139.4.1567 7789761 PMC1206485

[B44] Lee S. S. Lee R. Y. N. Fraser A. G. Kamath R. S. Ahringer J. Ruvkun G. (2003). A Systematic RNAi Screen Identifies a Critical Role for Mitochondria in *C. elegans* Longevity. Nat. Genet. 33, 40–48. 10.1038/ng1056 12447374

[B45] Mair W. Dillin A. (2008). Aging and Survival: The Genetics of Life Span Extension by Dietary Restriction. Annu. Rev. Biochem. 77, 727–754. 10.1146/annurev.biochem.77.061206.171059 18373439

[B46] Mantel N. (1966). Evaluation of Survival Data and Two New Rank Order Statistics Arising in its Consideration. Cancer Chemother. Rep. 50, 163–170. 5910392

[B47] Miller R. G. (1983). What Price Kaplan-Meier? Biometrics 39, 1077. 10.2307/2531341 6671119

[B48] Mudholkar G. S. Srivastava D. K. Kollia G. D. (1996). A Generalization of the Weibull Distribution with Application to the Analysis of Survival Data. J. Am. Stat. Assoc. 91, 1575–1583. 10.1080/01621459.1996.10476725

[B49] Petrascheck M. Miller D. L. (2017). Computational Analysis of Lifespan Experiment Reproducibility. Front. Genet. 8. 10.3389/fgene.2017.00092 PMC5492194 28713422

[B50] Phipson B. Smyth G. K. (2010). Permutation P-Values Should Never Be Zero: Calculating Exact P-Values when Permutations Are Randomly Drawn. Stat. Appl. Genet. Mol. Biol. 9, 39. 10.2202/1544-6115.1585 21044043

[B51] Pitt J. N. Strait N. L. Vayndorf E. M. Blue B. W. Tran C. H. Davis B. E. M. (2019). WormBot, an Open-Source Robotics Platform for Survival and Behavior Analysis in *C. elegans* . GeroScience 41, 961–973. 10.1007/s11357-019-00124-9 31728898 PMC6925079

[B52] Podshivalova K. Kerr R. A. Kenyon C. (2017). How a Mutation that Slows Aging Can Also Disproportionately Extend End-Of-Life Decrepitude. Cel Rep. 19, 441–450. 10.1016/j.celrep.2017.03.062 PMC5526670 28423308

[B53] Pollard J. H. Valkovics E. J. (1992). The Gompertz Distribution and its Applications. Genus 48, 15–28. 12286604

[B54] Puchalt J. C. Sánchez-Salmerón A.-J. Ivorra E. Llopis S. Martínez R. Martorell P. (2021). Small Flexible Automated System for Monitoring *Caenorhabditis elegans* Lifespan Based on Active Vision and Image Processing Techniques. Sci. Rep. 11. 10.1038/s41598-021-91898-6 PMC8192789 34112931

[B55] R Core Team R. (2015). A Language and Environment for Statistical Computing. Available at: https://www.r-project.org/ .

[B56] Rahman M. Edwards H. Birze N. Gabrilska R. Rumbaugh K. P. Blawzdziewicz J. (2020). NemaLife Chip: a Micropillar-Based Microfluidic Culture Device Optimized for Aging Studies in Crawling *C. elegans* . Sci. Rep. 10. 10.1038/s41598-020-73002-6 PMC7530743 33004810

[B57] Robinson A. (2007). Randomization, Bootstrap and Monte Carlo Methods in Biology. J. R. Stat. Soc A 170, 856. 10.1111/j.1467-985x.2007.00485_5.x

[B58] Samuelson A. V. Carr C. E. Ruvkun G. (2007a). Gene Activities that Mediate Increased Life Span of *C. elegans* Insulin-like Signaling Mutants. Genes Dev. 21, 2976–2994. 10.1101/gad.1588907 18006689 PMC2049198

[B59] Samuelson A. V. Klimczak R. R. Thompson D. B. Carr C. E. Ruvkun G. (2007b). Identification ofCaenorhabditis elegansGenes Regulating Longevity Using Enhanced RNAi-Sensitive Strains. Cold Spring Harbor Symposia Quantitative Biol. 72, 489–497. 10.1101/sqb.2007.72.068 18419309

[B60] Shaw W. M. Luo S. Landis J. Ashraf J. Murphy C. T. (2007). The *C. elegans* TGF-β Dauer Pathway Regulates Longevity *via* Insulin Signaling. Curr. Biol. 17, 1635–1645. 10.1016/j.cub.2007.08.058 17900898 PMC3124252

[B61] Stroustrup N. Anthony W. E. Nash Z. M. Gowda V. Gomez A. López-Moyado I. F. (2016). The Temporal Scaling of *Caenorhabditis elegans* Ageing. Nature 530, 103–107. 10.1038/nature16550 26814965 PMC4828198

[B62] Stroustrup N. Ulmschneider B. E. Nash Z. M. López-Moyado I. F. Apfeld J. Fontana W. (2013). The caenorhabditis Elegans Lifespan Machine. Nat. Methods 10, 665–670. 10.1038/nmeth.2475 23666410 PMC3865717

[B63] Stuhr N. L. Curran S. P. (2020). Bacterial Diets Differentially Alter Lifespan and Healthspan Trajectories in *C. elegans* . Commun. Biol. 3, 653. 10.1038/s42003-020-01379-1 33159120 PMC7648844

[B64] Sulston J. E. Horvitz H. R. (1977). Post-embryonic Cell Lineages of the Nematode, *Caenorhabditis elegans* . Dev. Biol. 56, 110–156. 10.1016/0012-1606(77)90158-0 838129

[B65] Tarone R. E. (1981). On the Distribution of the Maximum of the Logrank Statistic and the Modified Wilcoxon Statistic. Biometrics 37, 79. 10.2307/2530524

[B66] Therneau T. M. (2021). A Package for Survival Analysis in R. R Package. version 3.2-12. Available at: https://cran.r-project.org/package=survival .

[B67] Urban N. D. Cavataio J. P. Berry Y. Vang B. Maddali A. Sukpraphrute R. J. (2021). Explaining Inter-lab Variance in *C. elegans* N2 Lifespan: Making a Case for Standardized Reporting to Enhance Reproducibility. Exp. Gerontol. 156, 111622. 10.1016/j.exger.2021.111622 34793939 PMC8938996

[B68] Van Raamsdonk J. M. Hekimi S. (2011). FUdR Causes a Twofold Increase in the Lifespan of the Mitochondrial Mutant Gas-1. Mech. Ageing Development 132, 519–521. 10.1016/j.mad.2011.08.006 PMC4074524 21893079

[B69] Vanfleteren J. R. De Vreese A. Braeckman B. P. (1998). Two-Parameter Logistic and Weibull Equations Provide Better Fits to Survival Data from Isogenic Populations of *Caenorhabditis elegans* in Axenic Culture Than Does the Gompertz Model. Journals Gerontol. Ser. A: Biol. Sci. Med. Sci. 53A, B393–B403. 10.1093/gerona/53A.6.B393 9823735

[B70] Wilson D. L. (1994). The Analysis of Survival (Mortality) Data: Fitting Gompertz, Weibull, and Logistic Functions. Mech. Ageing Dev. 74, 15–33. 10.1016/0047-6374(94)90095-7 7934205

[B71] Winsor C. P. (1932). The Gompertz Curve as a Growth Curve. Proc. Natl. Acad. Sci. U.S.A. 18, 1–8. 10.1073/pnas.18.1.1 16577417 PMC1076153

[B72] Yon G. Marjoram P. (2019). slurmR: A Lightweight Wrapper for HPC with Slurm. Joss 4, 1493. 10.21105/joss.01493 PMC9432860 36052293

[B73] Zhao Y. Zhang B. Marcu I. Athar F. Wang H. Galimov E. R. (2021). Mutation Ofdaf‐2extends Lifespan *via* Tissue‐specific Effectors that Suppress Distinct Life‐limiting Pathologies. Aging Cell 20. 10.1111/acel.13324 PMC7963334 33609424

[B74] Zhigang Zhang Z. Jianguo Sun J. (2010). Interval Censoring. Stat. Methods Med. Res. 19, 53–70. 10.1177/0962280209105023 19654168 PMC3684949

