# Supplementary figures and images for: The Replica Set Method is a Robust, Accurate, and High-Throughput Approach for Assessing and Comparing Lifespan in C. elegans Experiments
Source: Front Aging. 2022 Apr 28;3:861701. doi: 10.3389/fragi.2022.861701 (PMC9261357; doi:10.3389/fragi.2022.861701)

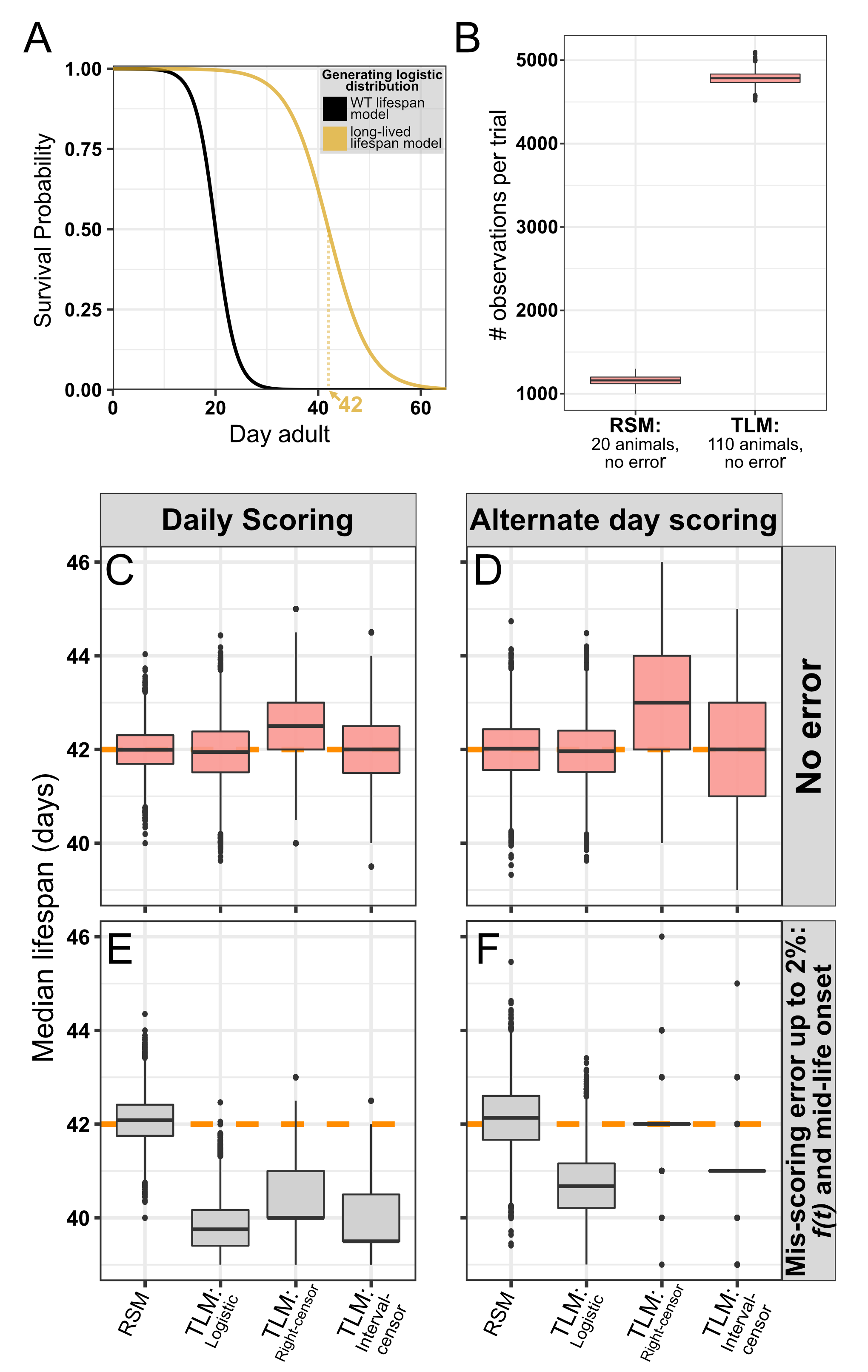

Supplement: Supplementary file 1 [file Image3.TIFF]

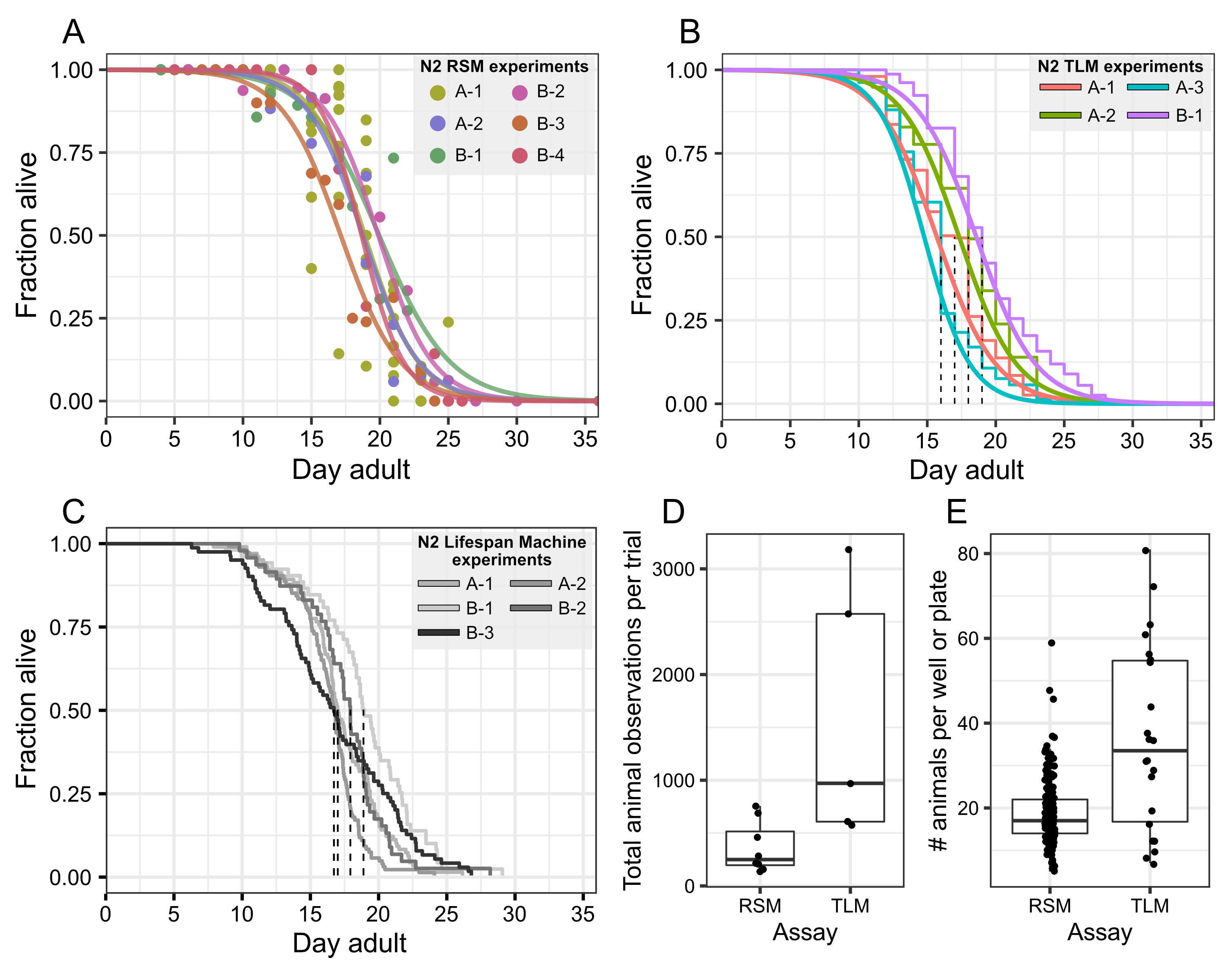

Supplement: Supplementary file 3 [file Image1.TIFF]

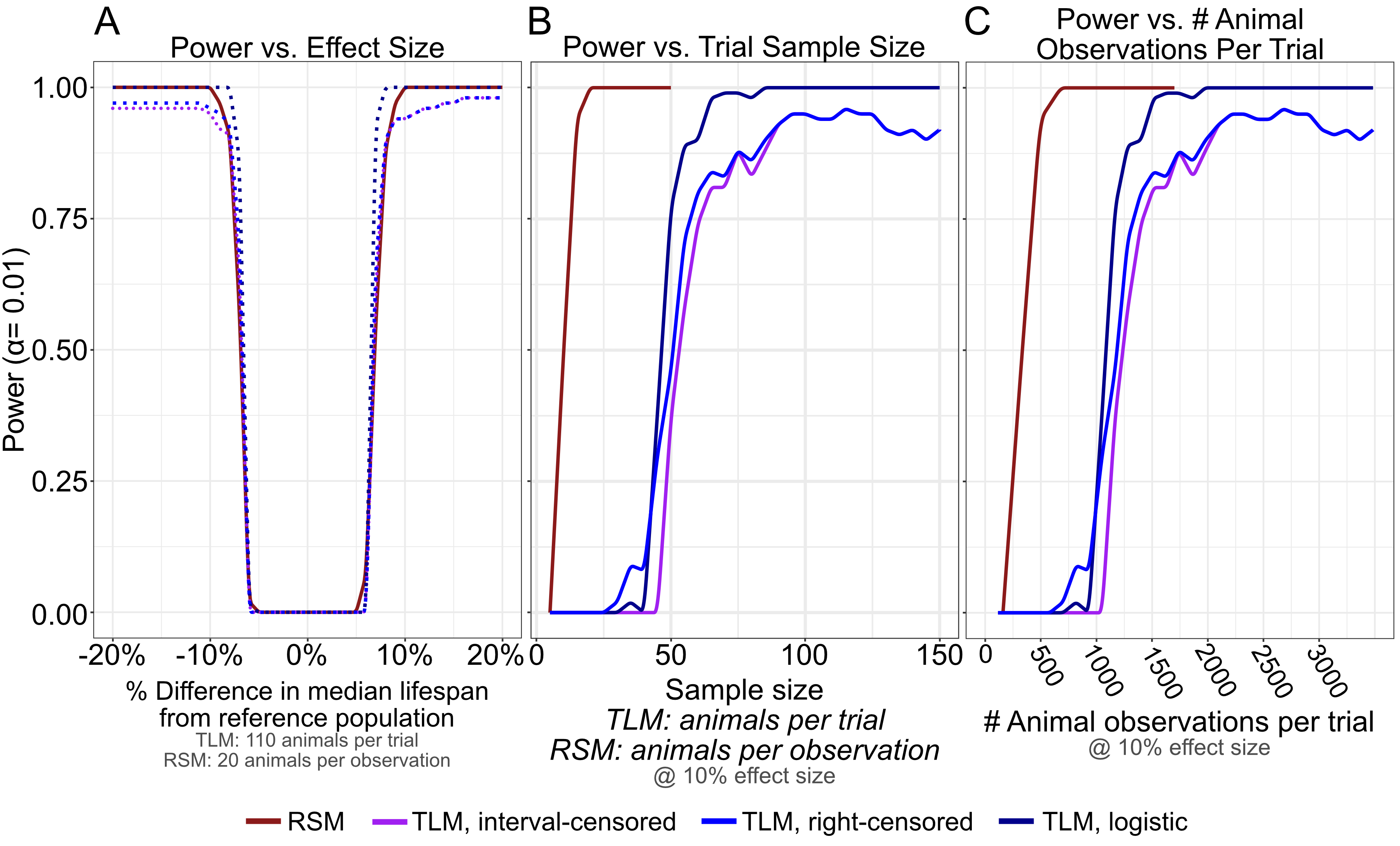

Supplement: Supplementary file 6 [file Image2.TIFF]
